# A Supramolecular Catalyst Self-Assembled From Polyoxometalates and Cationic Pillar[5]arenes for the Room Temperature Oxidation of Aldehydes

**DOI:** 10.3389/fchem.2018.00457

**Published:** 2018-10-16

**Authors:** Mengyan Zeng, Kun Chen, Junyan Tan, Jie Zhang, Yongge Wei

**Affiliations:** ^1^Key Lab of Organic Optoelectronics & Molecular Engineering of Ministry of Education, Department of Chemistry, Tsinghua University, Beijing, China; ^2^Beijing National Lab for Molecular Sciences, Key Lab of Polymer Chemistry and Physics of Ministry of Education, College of Chemistry and Molecular Engineering, Peking University, Beijing, China

**Keywords:** pillar[5]arenes, chromium centered Anderson polyoxometalates, nanospheres, supramolecular catalyst, aldehyde oxidation

## Abstract

Oxidizing aldehydes to generate carboxylic acids is a crucial reaction in nature and in chemical industry. The aldehyde oxidation, an easily achieved process in liver cells, is inert toward autoxidation in industrial production and difficultly achieved under enzymatic condition (in water, at pH 7, at room temperature). Herein, we prepared a supramolecular catalyst which are nanospheres assembled in aqueous media by chromium centered Anderson polyoxometalates Na_3_[CrMo_6_O_18_(OH)_3_] (namely, CrMo_6_) and cationic pillar[5]arenes (namely, P5A) with 10 positive charges which can be used as the phase transfer catalysts (PTCs). This supramolecular catalyst was exploited on aldehydes oxidation under enzymatic condition with relatively good conversion. Through DLS monitoring, the diameters of nanospheres were variable while changing the charge ratios of the ionic complexes (P5A-CrMo_6_), and it is probably because of the closer charge ratios causing the more compact assemblies. Also, the nano-morphologies were monitored by TEM and SEM, and the nanostructures were characterized by zeta potential, the X-ray energy-dispersive spectroscopy (EDS), elemental analysis.

## Introduction

Rational design of functional nanobuilding blocks with well-defined nanostructure and specific functionality is very important. Polyoxometalates (POMs) are well-defined early transition metal-oxygen anionic clusters with various chemical composition and nanosized architecture (Müller and Pope, 1991; Dolbecq et al., 2010; Miras et al., 2012). The potential applications range from catalysis (Mizuno et al., 2005), electronics (Kawasaki et al., 2011), magnetism (Poblet et al., 2003), and photochemistry(Li et al., 2011), to medicines(Rhule et al., 1998). POMs possesses the assembly ingredients for forming different kinds of nanostructures (Li et al., 2017). Spontaneous self-assembly of weak electrolyte type POMs into vesicle-like supramolecular structures has been confirmed by Liu et al. (Liu, 2002; Liu et al., 2003; Liu and Liu, 2005a,b). The assistance from grafting groups or counterions has been demonstrated to be more effective in constructing POM self-assemblies (Zhang et al., 2008; Landsmann et al., 2010; Yin et al., 2013; Zhu et al., 2013). Compared with the covalent modification of POM with grafting groups, utilization of electrostatic interactions between anionic POMs and cationic amphiphiles may be more versatile for the hierarchical self-assembly of POM clusters (Ishiba et al., 2017; Li et al., 2017; Cheng et al., 2018).

Pillararenes, as a new generation of macrocyclic host, with hydroquinone as units linked at para position, have attracted much attention since its first report by Ogoshi (Ogoshi et al., 2008). Compared with the other macrocycles such as crown ethers, cyclodextrin, calixarene, and cucurbituril, pillararenes possess a columnar shape with a p-electron rich cavity, having unique characteristics, such as easy and facile synthesis, various modification sites, pillar architectures, making them favorable building blocks in developing self-assembly for constructing vesicles, molecular machines, artificial transmembrane channels and so on (Xue et al., 2012; Tan and Yang, 2015; Ogoshi et al., 2016).

**Figure 1:**
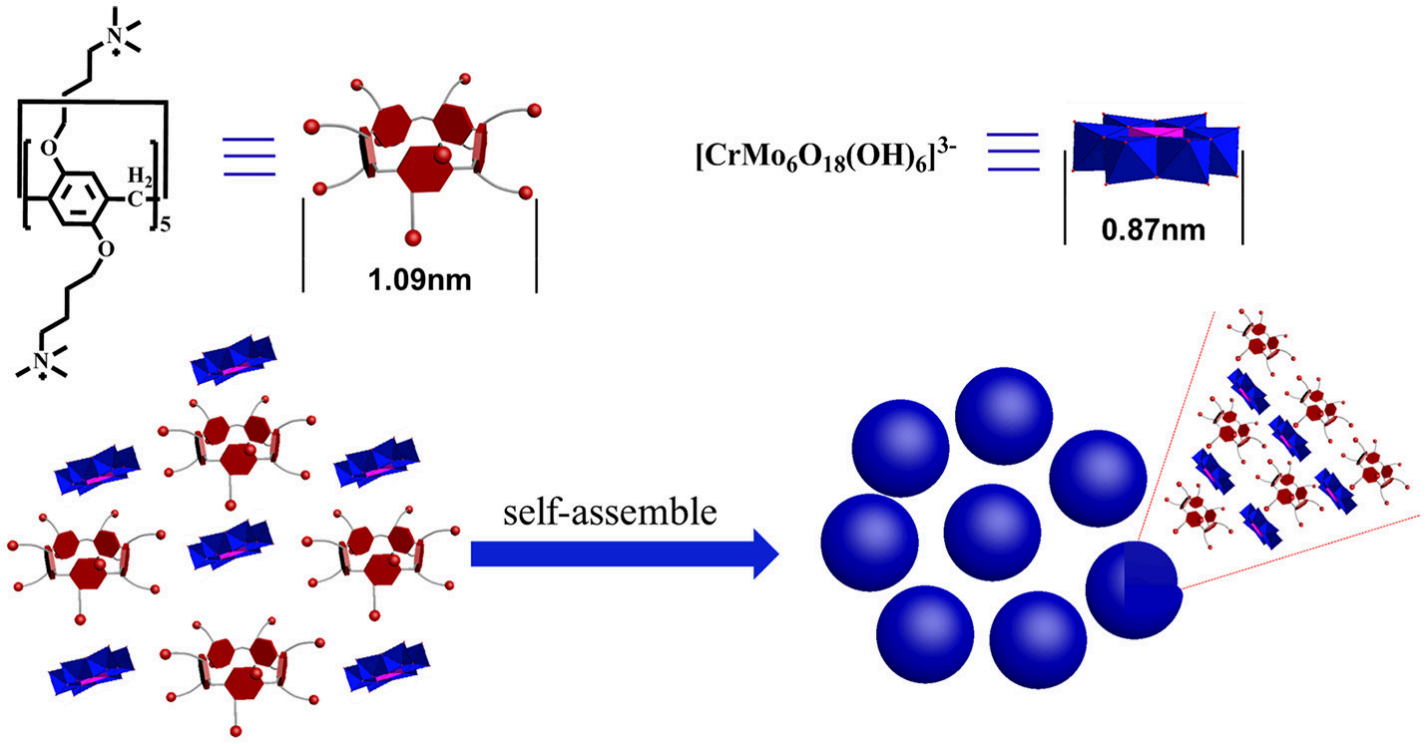
Schematic representation of the two chemical structures and their self-assemblies.

Oxidizing aldehydes to produce carboxylic acids is a crucial reaction in nature and in chemical industry (Latchman, 1995; Crabb et al., 2004). In living cells, oxidizing aldehydes into acids is conducted at body temperature using oxygen as oxidizing agent and dehydrogenase as the catalyst in neutral aqueous solution (Crabb et al., 2004). There are relatively few examples of models performing under enzymatic condition (in water, at pH 7, at room temperature) (Marinescu and Bols, 2006; Raynal et al., 2014). Generally, despite being prone to autoxidation, most kinds of aldehydes are relatively stable (Liu and Li, 2016; Zhang et al., 2017). The majority of these oxidation reactions in modern industry require stoichiometric amounts of hazardous oxidants such as KMnO_4_ (Kleiderer, 1930; Ruhoff, 1936), CrO_3_ (Sandborn, 1929), KHSO_5_ (Mo, 2006), KIO_4_ (Travis et al., 2003), etc., and often take place in organic solvents. Since Li et al. sequentially reported first homogeneous silver- and copper- catalysis of aerobic aldehyde oxidation in water (Liu et al., 2015; Liu and Li, 2016), Wei et al. reported a single-sided triol-functionalized iron centered Anderson POM catalysis (Yu et al., 2017). POMs combine high reactivity and stability in oxidation catalysis (Lechner et al., 2016). Phase transfer catalysts (PTCs) provide easy, inexpensive and versatile solutions for organic reactions (Maruoka and Ooi, 2003; Hashimoto and Maruoka, 2008). It accelerates the reaction rate by improving the solubility of the reactants isolated in different liquid phases. Herein, we used cationic pillar[5]arenes (namely, P5A) as PTCs and exploited the P5A-CrMo_6_ supramolecular complex (Figure 1) assembled by the chromium centered polyoxometalates Na_3_[CrMo_6_(OH)_3_] (namely, CrMo_6_) and P5A under aqueous conditions. When applied to catalyze aldehydes into carboxylic acids, this supramolecular catalysis (Leeuwen, 2008) offered better catalytic effect than CrMo_6_ or P5A alone with air as the oxidant in water under enzymatic conditions as a result of synergy effect that combined advantage of CrMo_6_ and P5A.

## Experimental section

### Materials and methods

Boron trifluoride diethyl etherate, and 1,4-Dibromo butane were purchased from Aladdin. All the other chemicals were purchased from Sinopharm Chemical Reagent Co., Ltd. All the reagents were used without further purification.

Na_3_[Cr(OH)_6_Mo_6_O_18_] were prepared according to previously reported procedures (Wu et al., 2011). Na_2_MoO_4_ (12 g, 57.5 mmol) was dissolved in water (10 mL). Keeping 80 °C, concentrated nitric acid (4.5 mL) was added to the solution. Then stop the heating equipment, and Cr(NO_3_)_3_ (3.2 g) was slowly added to the solution. When a large amount of red precipitates appeared in the solution, the reaction was stopped. After cooled down to room temperature, the solution was filtered, and 8.9 g of the red solid was collected as the product. Yield: 84% based on Mo.

The cationic water-soluble pillar[5]arene (P5A) were prepared according to previously reported procedures (Ma et al., 2011; Yao et al., 2012, 2014) (Supplementary Figure 1). ^1^H-NMR (400 MHz, D_2_O) δ (ppm) (Supplementary Figure 2): 6.73 (s, 10H), 3.80 (s, 30H), 3.10 (s, 20H), 2.91 (s, 90H), 1.59 (s, 40H). ^13^C-NMR (400 MHz, D_2_O) δ (ppm) (Supplementary Figure 3): 166.98, 150.21, 129.32, 66.13, 57.91, 52.91, 25.84, 19.54. HRESI-MS (Supplementary Figure 4): m/z calcd for [M−2Br]^2+^ 1196.36002; [M– 3Br]^3+^ 770.60389; [M−4Br]^4+^ 557.72439; [M−5Br]^5+^ 430.39624; [M−6Br]^6+^ 345.17750; [M−7Br]^7+^ 284.59296, [M−8Br]^8+^ 239.06875; [M−9Br]^9+^ 203.59118.

The preparation of the nanospheres were by mixing different charge ratios of cationic P5A and anionic CrMo_6_ in water solution, with the concentration of P5A and CrMo_6_ kept constant at 0.05 mg/mL.

**NMR:** ^1^H-NMR and ^13^C-NMR were performed on JNM-ECA400 equipment. **TEM:** TEM images were obtained on a JEMO 2010 Electron microscopy with an operational acceleration voltage of 120 kV. The samples were prepared by fishing the carbon coated copper grid into the aqueous solution and then dried in air at 25°C for 30 min. **HPLC:** HPLC was performed on a Waters 2695 with a UV detector and refractive index detector. The gradient elution was performed with 8 mM (NH_4_)_2_HPO_4_ at a rate of 0.7 mL/min. Fifty microliter of prepared sample or standard solution was injected. **HRESI-MS:** HRESI-MS was performed on a on a Flash EA 1112 full-automatic mass spectrometer, and the experiment was carried out in the positive-ion mode using CHCl_3_ as the solvent. **SEM:** SEM was performed on Hitachi SU-8010 Electron microscopy with an operational acceleration voltage of 200 V−50 KV. The preparation of samples was by dropping about 20 μL of the solution on a cleaved silicon surface. The gold spraying time is 30 seconds. **FT-IR:** IR was carried out on a Perkin Elmer Spectrum. The solid samples were prepared by vacuum drying at 50°C.

### Catalytic experiments

#### General procedure for the oxidation of aldehydes

Prepare 1.0 mM P5A, 1.0 mM CrMo_6_ aqueous solution, respectively. Pipette 43 μL P5A (1.0 mM), 144 μL CrMo_6_ (1.0 mM), and 4770 μL water into a PE tube. After ultrasonic bath for 1 min, the nanosphere (P5A-CrMo_6_) solids were collected though high speed centrifugation (14,000 rpm, 25°C, 5 min), washed with pure water, and purified by centrifugation. Then the collected colloids were dried at 50°C under vacuum to obtain the products. Dry weight: 0.2 mg. Yield: 81.1% based on P5A and CrMo_6_. The catalytic system contains P5A-CrMo_6_ (0.2 mg, 0.002 mmol, 0.2 mol %), benzaldehyde (101 μL, 1 mmol), air (using air pump to control the flow velocity), H_2_O (5 mL). The mixture was stirred at 30°C, and the reaction progress was monitored by HPLC. After 12 h, 3 × 5 mL ethyl ether was added by extracting three times to give the organic phase, and the solid catalyst was isolated by filtration. Then the organic phase was diluted 100 times by acetonitrile. The diluted samples were loaded into sample vials and detected by HPLC, using acetonitrile and water as mobile phase, non-polar C18 as separation column, UV and refractive index detector as detector.

## Results and discussion

**Figure 2:**
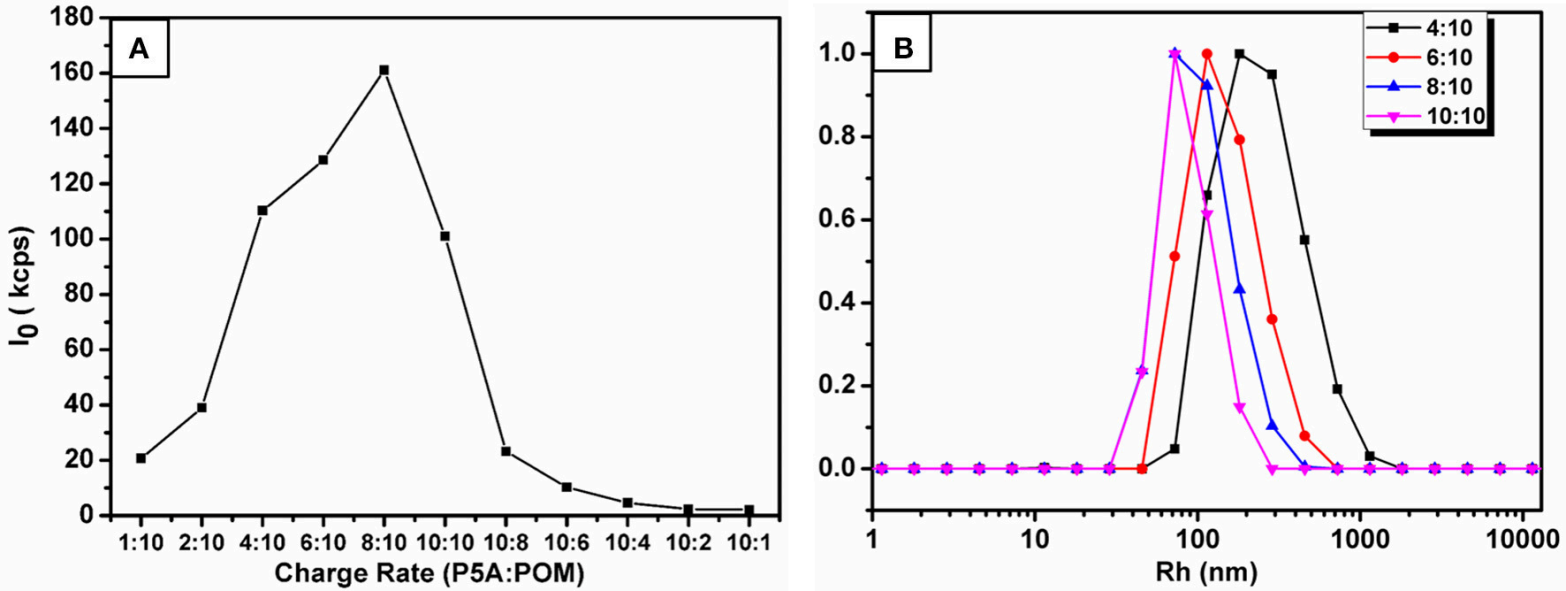
**(A)** DLS total scattered intensity ***I***of the P5A-CrMo_6_ complex at different charge ratios. From left to right, the charge rate of P5A:CrMo_6_ is 1:10, 2:10, 4:10, 6:10, 8:10, 10:10, 10:8, 10:6, 10:4, 10:2, 10:1. The concentration of (P5A-CrMo_6_) was controlled at 0.05 mg/mL. **(B)** The size distribution at different charge ratios obtained by a CONTIN analysis with normalized counts by DLS.

The supramolecular amphiphiles (SA) (Wang et al., 2010; Han et al., 2011; Zhang and Wang, 2011) was prepared by mixing different charge ratios of cationic P5A and anionic CrMo_6_ in water solution, with the concentration of P5A and CrMo_6_ kept constant at 0.05 mg.mL^−1^. After the samples were mixed, the assembling process took place rapidly. Dynamic light scattering (DLS) was used to monitor size evolution of these assemblies with different charge ratios. We define the compositional formation of the charge ratio (r) between P5A and CrMo_6_, *r* = (3 P5A^10+^): (10 CrMo${}_{6}^{3-}$). The DLS count rates, shown in Figure 2A, were very low until r reached 0.4. When 0.4 ≤ *r* ≤ 1.0, the DLS count rates were more than 100 kcps. But when 1.0 < *r*, the DLS count rates were also low (even less than 10 kcps), indicating no detectable assemblies in the solution. When *r* < 0.4, the charge ratio of P5A and CrMo_6_ is greatly unmatched, and the size of assembles is big and thermodynamically unstable. A small amount of precipitates was found at the bottom of the bottle in a very short time. That is why the DLS count rate is low when *r* < 0.4. As the charge ratios of P5A and CrMo_6_ became closer, the hydrodynamic radii (*R*_h_) of assemblies in aqueous solution become smaller, because of the more compact assembly due to the closer charge ratios. Average size of the nanospheres was obtained by dynamic light scattering, shown in Figure 2B. For charge ratios at 4:10, 6:10, 8:10, 10:10, the hydrodynamic radius is 567, 295, 130, and 75 nm, respectively. Combined with relative TEM images, shown in Supplementary Figure 5, the closer charge ratios caused the more compact assembles. However, for *r* > 1.0, these solutions were clear during the 17 days of testing time, and almost no regular aggregates can be observed for TEM testing, which indicated that there were no convinced assembles. It is convinced that the size of these aggregates are molar ratios dependently.

**Figure 3:**
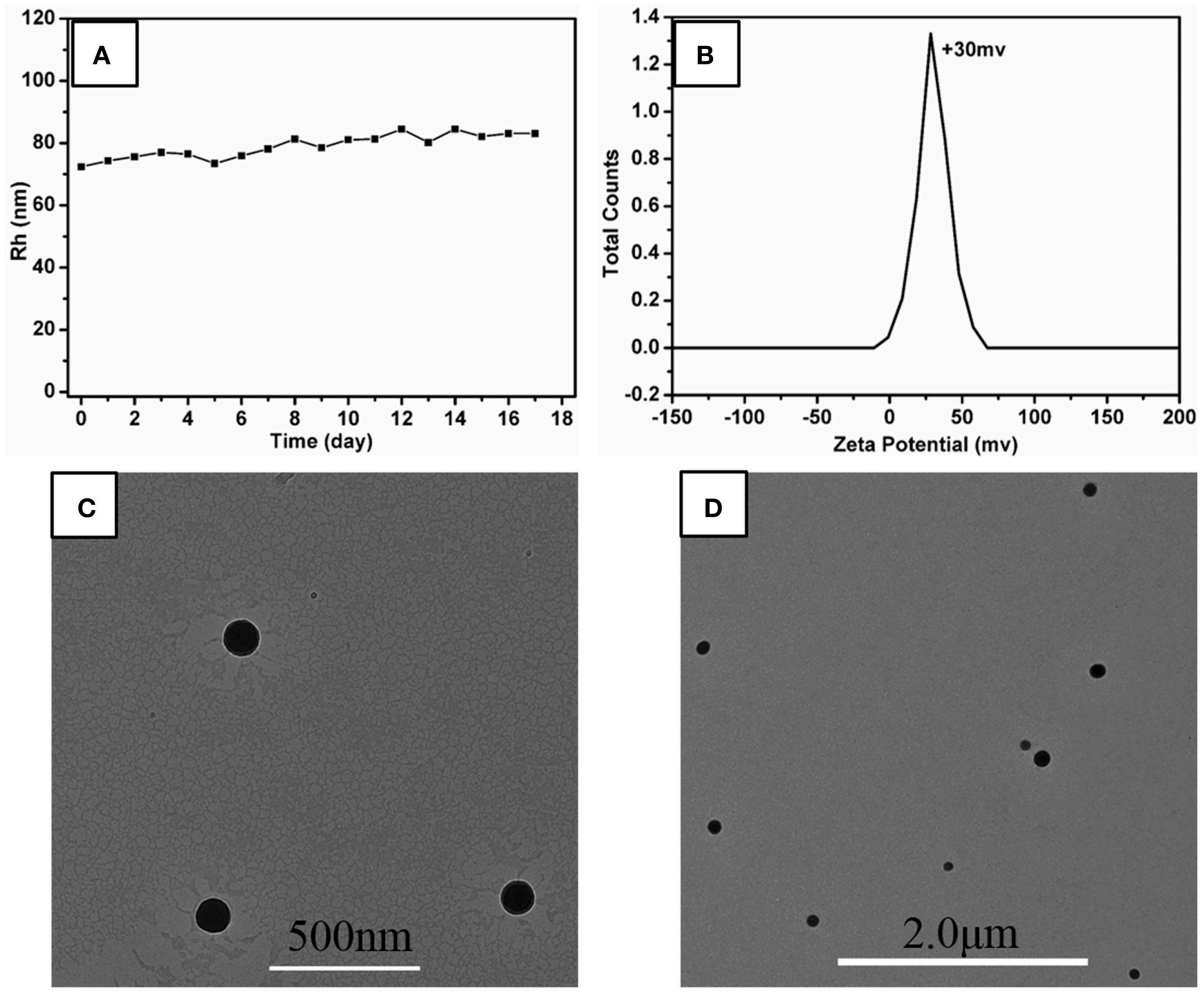
**(A)** Change of hydrodynamic radii (*R*_*h*_) of P5A-CrMo_6_ in water solution with time. Charge ratio of P5A-CrMo_6_ is 10:10. The concentration of P5A-CrMo_6_ is 0.05 mg/mL. **(B)** Zeta potential of the P5A-CrMo_6_ self-assemblies with charge ratio 10:10 at 0.05 mg/mL in water solution. **(C)** TEM image of the self-assemblies immediately after mixed together. **(D)** TEM images of self-assemblies after 17th day.

In this investigation, the system of *r* = 10:10 was chose as the research object, named P5A-CrMo_6_, which has the highest DLS count rate and thermodynamically stable R_h_ at ~75 nm, shown in Figure 2B. The hydrodynamics radius of the aggregates in the aqueous solution slowly increased over time, shown in Figure 3A. During the first 17 days, there is still no precipitate in the solution, which confirms the stability of the aggregates. The hydrodynamic radii of nanospheres grows with time. The average hydrodynamic radii became bigger from 75 nm on first day to 87 nm on the 17th day, as shown in Figure 3A. This is because more and more compounds (POM or P5A) aggregated on the outer surface of the nanospheres, as time goes by. And the nanospheres diameters of the 1st day, the 8th day, the 17th day were also counted from TEM images (Figures 3C,D and Supplementary Figures 6A,C,E). By analyzing the size-dispersion histogram of nanospheres (Supplementary Figures 6B,D,F), the average diameters are 99, 111, and 150 nm, respectively. Compared with the DLS data, all the statistical sizes of the nanospheres in TEM images are a litter smaller which is directly caused by the removing hydration layer of nanospheres under drying (Supplementary Figure 7). Furthermore, X-ray energy-dispersive spectroscopy (EDS) coupled with HRTEM shows that both molybdenum, chromium, bromine, nitrogen, and oxygen elements exist throughout the assemblies, shown in Supplementary Figure 8. The molybdenum and chromium elements are attributed to CrMo_6_, and the bromine and nitrogen elements are attributed to P5A.

In order to explore the assembly structure, scanning electron microscopy (SEM) and zeta potential were taken. As shown in Supplementary Figure 9, the sphere aggregates were solid structure. And the zeta potential of the aggregates was determined to be +30 mV, shown in Figure 3B, indicating that the outer surface of these aggregates is covered by cations, probably the cationic P5A. The composition of these nanospheres determined by elemental analysis was CrMo_6_:P5A = 3.3:1, which indicated that the CrMo_6_ anion is surrounded by P5A in the form of P5A·(CrMo_6_)_3.3_, shown in Supplementary Table S1. The structure was still maintained as supported by FTIR spectra, shown in Supplementary Figure 10. The peaks at 908 cm^−1^ n (Mo-O_b_-Mo) and 945 cm^−1^ n (Mo-O_d_, O_d_ = terminal oxygen) are consistent with the structure of CrMo_6_, indicating the Anderson-type structure is maintained during assembling. Herein, the diameter of P5A (10.0 Å) by DFT calculation is larger than CrMo_6_ (8.7 Å). The surface occupation of CrMo_6_ is greater than 2/3. As a result, no classical reverse bilayer can be formed (Volkmer et al., 2000; Bu et al., 2002, 2005; Qi et al., 2008; Li et al., 2017). The tight packing of rigid balls instead of classical reverse bilayer were the probably formed state. It is consistent with solid aggregates detected by SEM and TEM. The assembled structure assumed be arranged as shown in Figure 1.

**Figure 4:**
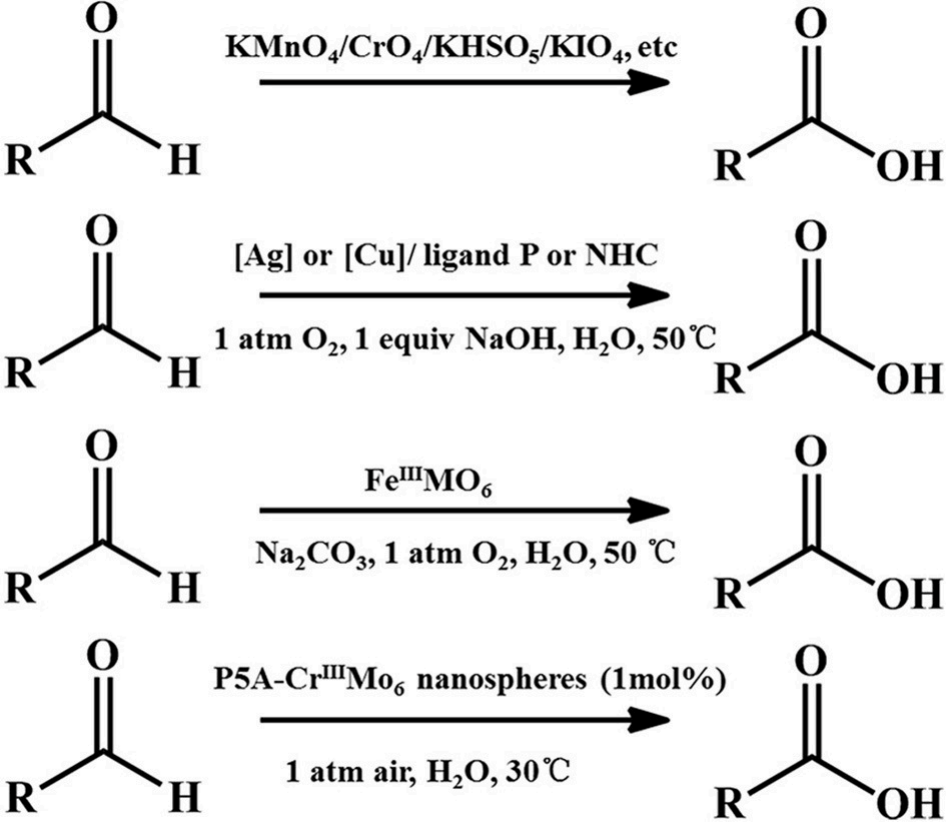
Representative examples of oxidation of aldehydes.

**Figure 5:**
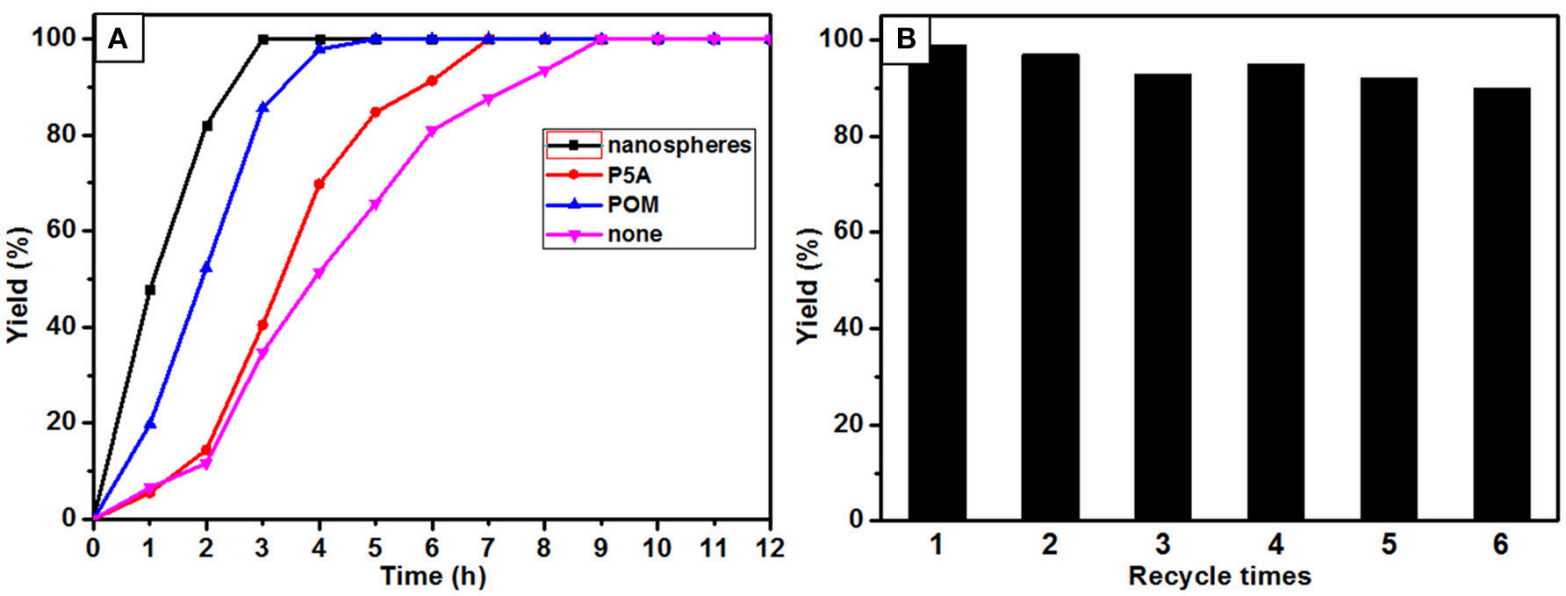
Reaction dynamics **(A)** and catalytic experiment of recycled of nanospheres **(B)**.

Catalytic oxidations of aldehydes into carboxylic were investigated to further explore the function of the P5A-CrMo_6_ nanospheres, shown in Figure 4. Air as oxidant was simply flushed into the reaction flask (with a balloon) under aqueous reaction conditions. Through optimizing the catalytic reaction temperature conditions, we chose 30°C as the reaction temperature. Taking catalytic oxidation benzaldehyde to benzoic acid as the key research example, the reaction kinetics was monitored by liquid chromatography at different time points, shown as Figure 5A. For nanospheres, after 3 h, the yield of benzoic acid has reached more than 96%. According to the control experiments, when the catalyst is Anderson-type POMs Na_3_[CrMo_6_O_18_(OH)_3_] alone, the yield reaching 90% needs at least 4 h. When only cationic P5A is used as catalyst, reaching above 90% of yield needs at least 6 h. Since the aldehydes oxidation is an auto-oxidation process, when there is no catalyst, the benzaldehyde can also be oxidized, however, reaching above 90% yield needs at least 8 h. In general, most kinds of aldehydes are stable, despite being prone to autoxidation, requiring a long reaction time. Water, the most environmental benign solvent, was chosen as the solvent in this oxidation process. Unfortunately, most of aldehydes can't be dissolved in water, for example, the lauric aldehyde. The cationic pillararenes are a new kind of phase transfer catalysis similar to TBABr, which can drag the insoluble organic aldehyde into water. It is the key factor to improve the contact area between catalyst and substrate. As heterogeneous catalysts, the nanospheres have the synergistic catalytic effect of Anderson type POM CrMo6 and organic cationic P5A which acts as phase transfer catalyst.

**Figure 6:**
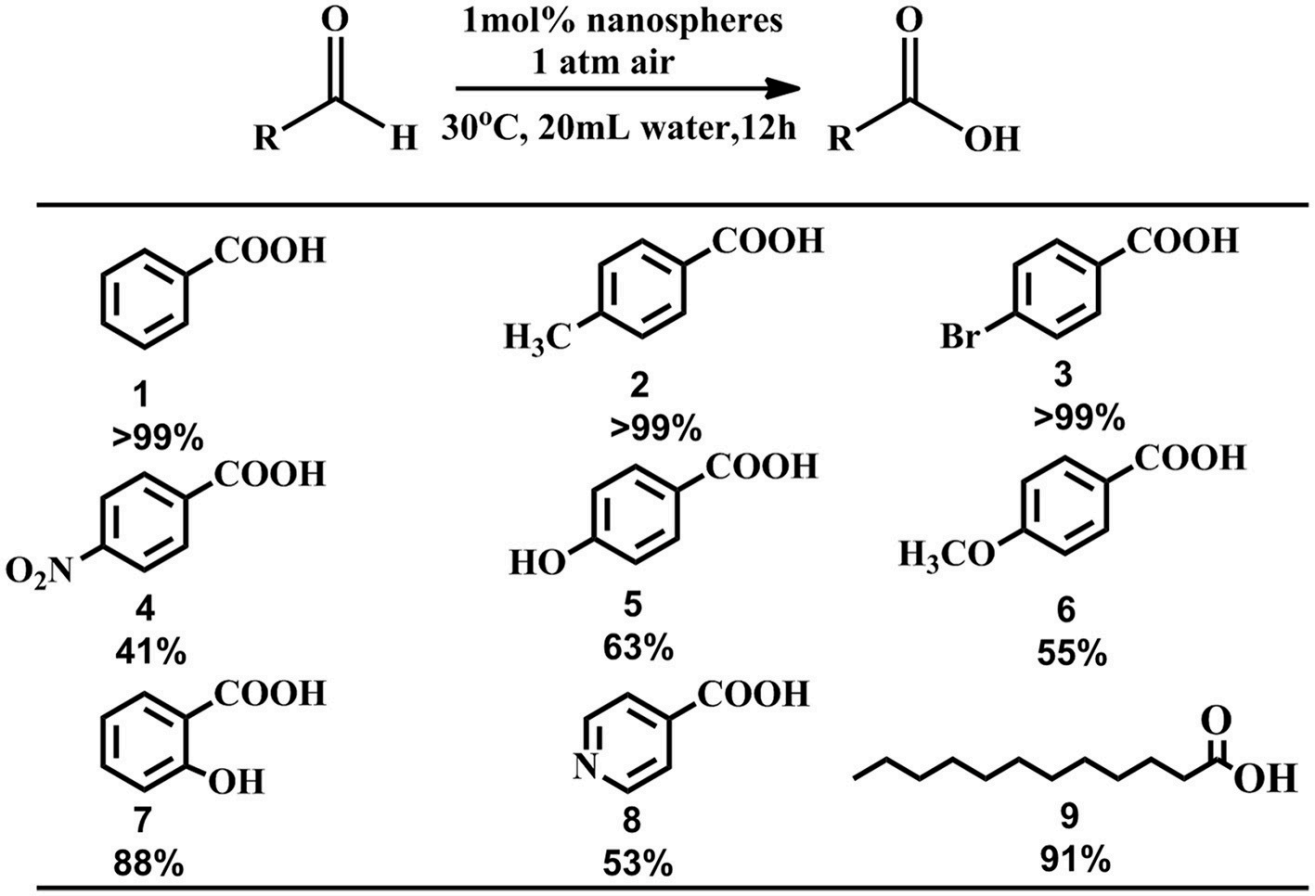
Investigation of substrate scope.

In order to explore the scope and functional-group compatibility of these nanospheres, a number of functionalized aldehydes were used as substrates to carry out the reaction under the optimized conditions, shown in Figure 6. Aromatic aldehyde bearing a methyl group was oxidized in quantitative yields above 99% (products **2**) after 12 h. Halogen-substituted aldehydes such as 4-bromobenzaldehyde were tolerated under the optimized reaction conditions and the corresponding carboxylic acids were obtained in quantitative yields above 99% (products **3**). However, when aromatic aldehydes bearing electron-withdrawing groups such as 4-nitrobenzaldehydes, 4-hydroxybenzaldehyde, 2-hydroxybenzaldehyde, 4-methoxybenzaldehyde, the obtained oxidized carboxylic acid yields are low (products **4**–**7**). Because of the water-solubility of 4-hydroxybenzaldehyde and 2-hydroxybenzaldehyde, their oxidation yields are relatively higher compared with 4-nitrobenzaldehyde and 4-methoxybenzaldehyde. Heterocyclic aromatic aldehyde such as 4-pyridinecarboxaldehyde was also oxidized with a yield about 53% (products **8**). The aliphatic aldehyde such as lauric aldehyde was also tested. Interestingly, the oxidation yield is surprisingly high, with an essentially quantitative yield of 91% (products **9**). The strong host-guest interaction between the cationic pillar[5]arenes and lauric aldehyde or lauric acid might result in water-insoluble lauric aldehyde penetrating the positive pillar[5]arenes because of electrostatic interaction, hydrogen bond interaction and CH/π interaction, and then lauric aldehyde might be more susceptible to be oxidized.

The recycling and stability of the nanosphere catalysts were also evaluated. After six cycles, the catalytic yield of nanospheres is still above 80%, shown in Figure 5B. The structure of the catalyst is basically unchanged after six reaction cycles as confirmed by FT-IR spectrum, shown in Supplementary Figure 10. Unfortunately, the spherical aggregates unable to maintain their original morphology after the second cycle of experiments, but the aggregates are still kept in nanosized, shown in Supplementary Figure 11. This result explained why the catalytic activity still maintained after six cycles.

## Conclusion

In summary, we exploited the co-assembly of polyanionic functional chromium centered POM [CrMo_6_O_18_(OH)_3_]^3−^ and polycationic functional pillar[5]arenes in aqueous solution. Interestingly, the nanospheres were formed and their diameters were variable along with the changing of charge ratios of CrMo_6_:P5A in ionic complexes. The regularity of the assembly of different charge ratio was detected through DLS, and the morphology of these nanospheres were observed by SEM and TEM. Combined with zeta potential, we presumed a structural model to this system. The well-defined nanospheres were explored as the catalyst for catalytic oxidation of aldehydes into carboxylic acids. Synergy effect which combined both advantages of [CrMo_6_O_18_(OH)_3_]^3−^ and P5A offers better catalytic effect with relatively good conversion. Future directions of this work are aimed at continuing to find amazing nanostructures, probing the mechanism and exploiting different applications.

## Author contributions

MZ did most experiments and data collection work, and wrote the manuscript. KC helped some key data analysis and revised the manuscript. JT helped to do the DLS experiments and revise the manuscript. JZ and YW guided the entire experiments and revised the manuscript.

### Conflict of interest statement

The authors declare that the research was conducted in the absence of any commercial or financial relationships that could be construed as a potential conflict of interest.
