# Supplementary material for: A Supramolecular Catalyst Self-Assembled From Polyoxometalates and Cationic Pillar[5]arenes for the Room Temperature Oxidation of Aldehydes
Source: Front Chem. 2018 Oct 16;6:457. doi: 10.3389/fchem.2018.00457 (PMC6198131; doi:10.3389/fchem.2018.00457)
Supplement: Supplementary file 1 [file Data_Sheet_1.docx]

Supplementary Material

A Supramolecular Catalyst Self-assembled from Polyoxometalates and Cationic Pillar[5]arenes for the Room Temperature Oxidation of Aldehydes

Mengyan Zeng^1#^, Kun Chen^1#^, Junyan Tan^2^, Jie Zhang^2*^, Yongge Wei^1*^

^1^ Key Lab of Organic Optoelectronics & Molecular Engineering of Ministry of Education, Department of Chemistry, Tsinghua University, Beijing, P.R. China

^2^ Beijing National Lab for Molecular Sciences, Key Lab of Polymer Chemistry and Physics of Ministry of Education, College of Chemistry and Molecular Engineering, Peking University, Beijing, P. R. China.

*** Correspondence:**

Yongge Wei

[yonggewei@tsinghua.edu.cn](mailto:yonggewei@tsinghua.edu.cn)

Jie Zhang

[jz10@pku.edu.cn](mailto:jz10@pku.edu.cn)

# Supplementary Figures and Tables


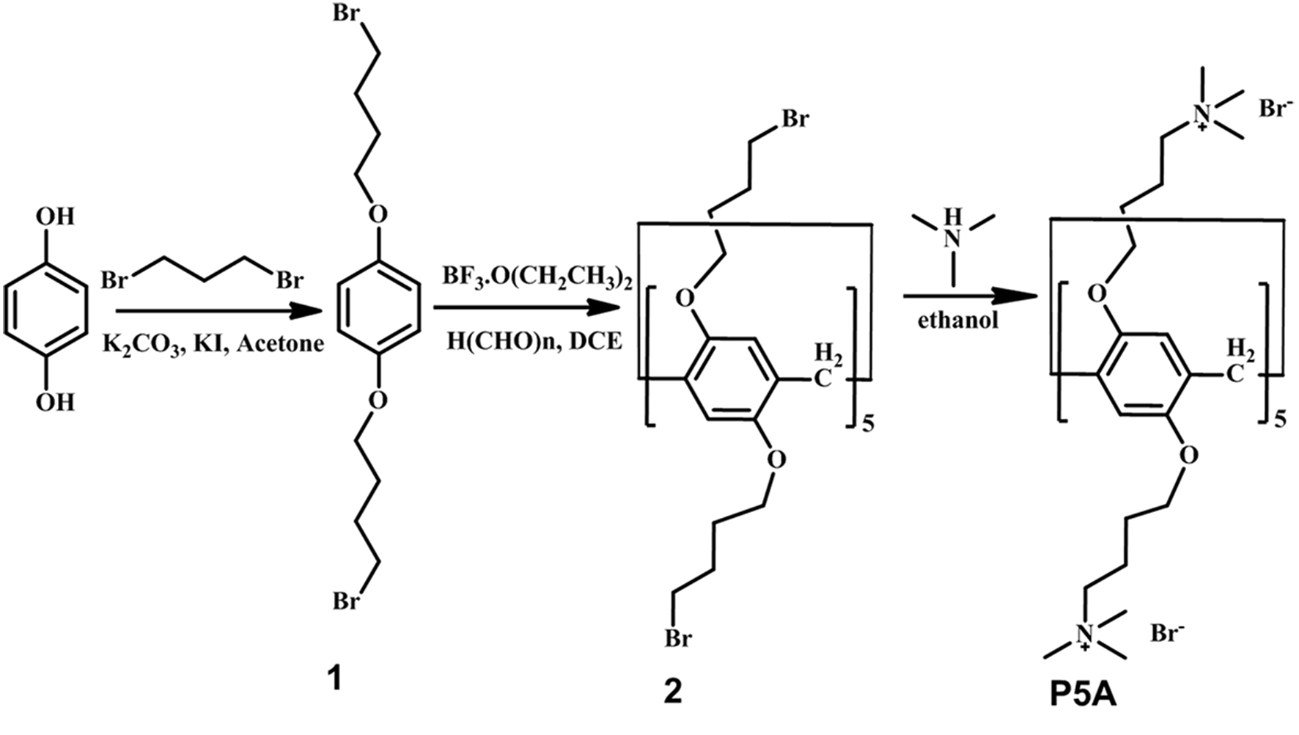


**Supplementary Figure 1.** Synthetic route for the cationic water-soluble pillar[5]arene.


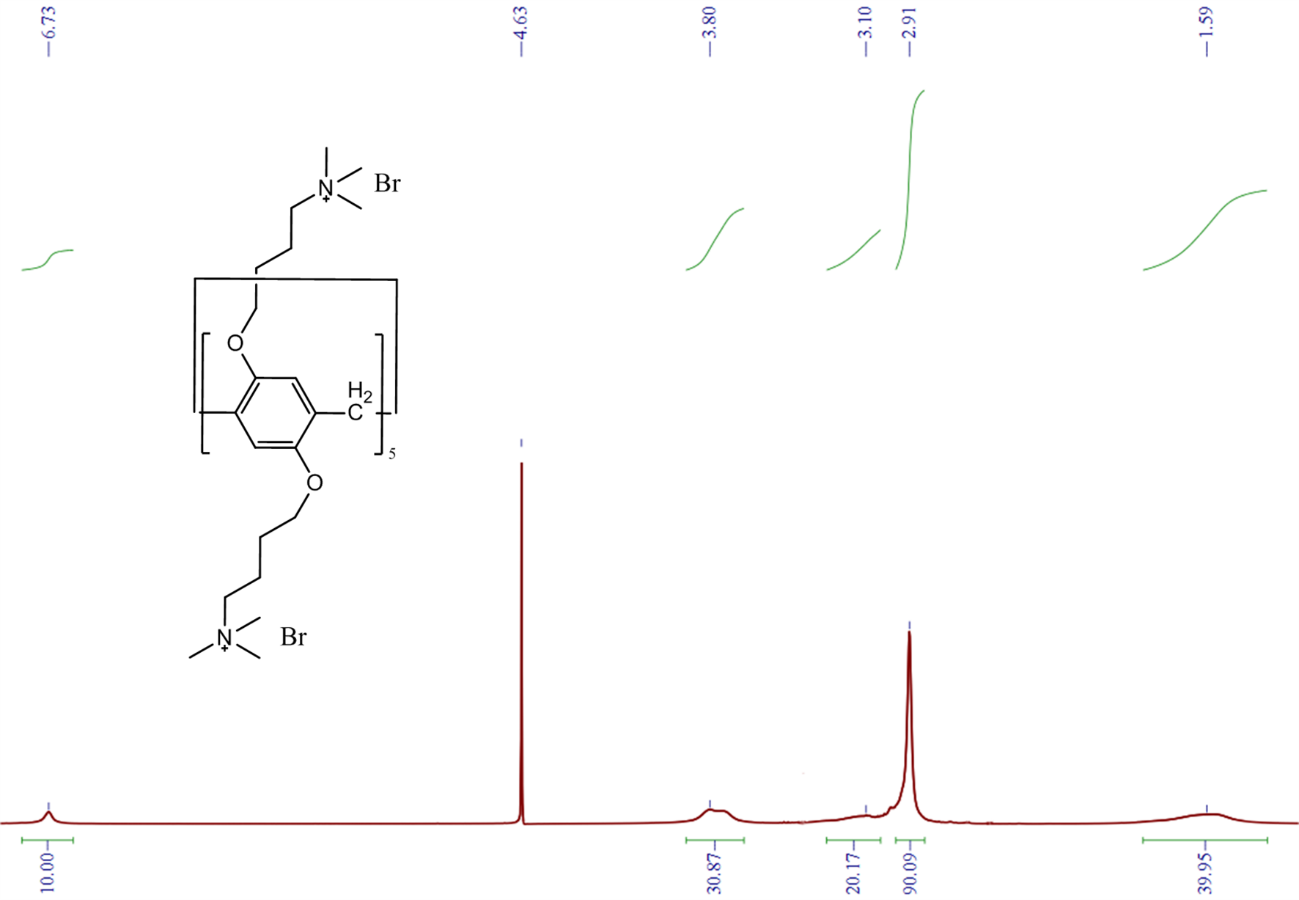


**Supplementary Figure 2.** ^1^H-NMR spectrum (400 MHz) of P5A in D_2_O. ^1^H-NMR (400 MHz, D_2_O) δ (ppm): 6.73 (s, 10H), 3.80 (s, 30H), 3.10 (s, 20H), 2.91 (s, 90H), 1.59 (s, 40H).


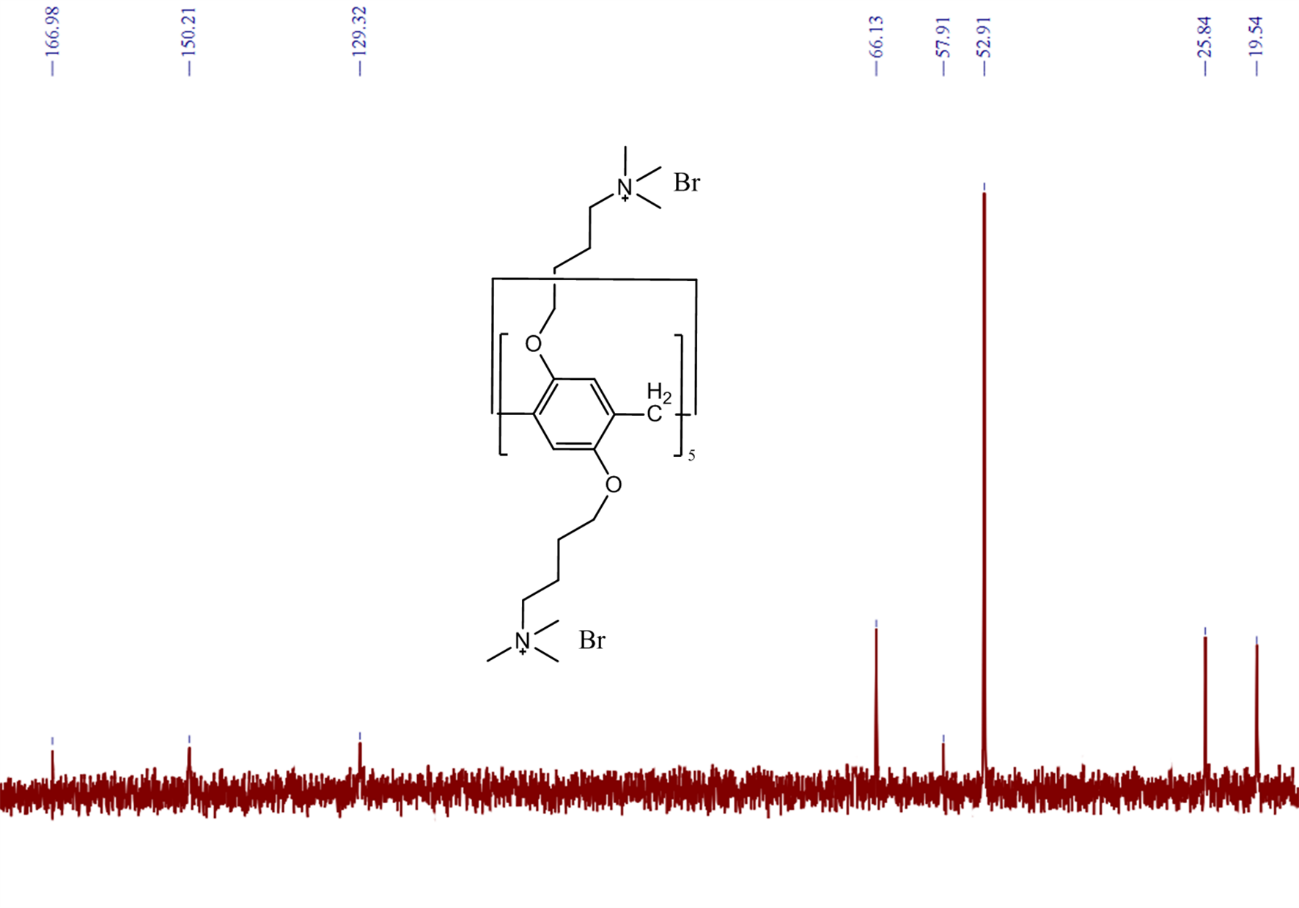


**Supplementary Figure 3.** ^13^C-NMR spectrum (400 MHz) of P5A in D_2_O. ^13^C-NMR (400 MHz, D_2_O) δ (ppm): 166.98, 150.21, 129.32, 66.13, 57.91, 52.91, 25.84, 19.54.


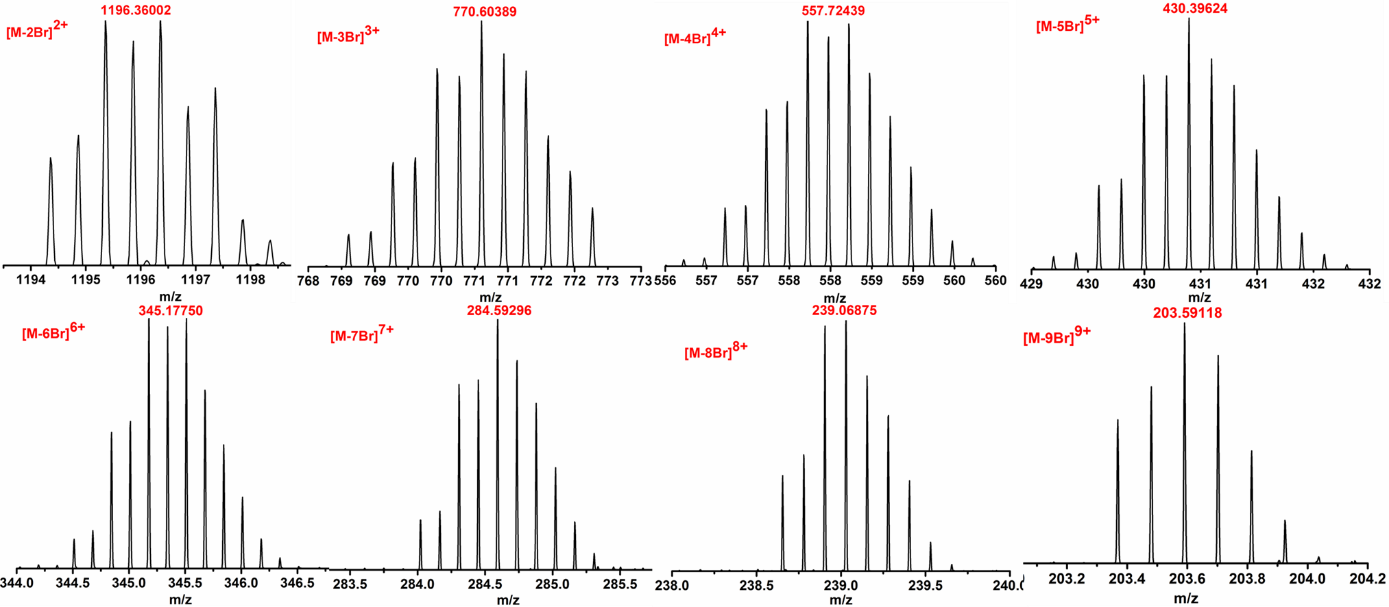


**Supplementary Figure 4.** High resolution electrospray ionization mass spectrum of cationic P5A. HRESI-MS is shown in Fig S3: m / z calcd for [M-2Br]^2+^ 1196.36002; [M-3Br]^3+^ 770.60389; [M-4Br]^4+^ 557.72439; [M-5Br]^5+^ 430.39624; [M-6Br]^6+^ 345.17750; [M-7Br]^7+^ 284.59296, [M-8Br]^8+^ 239.06875; [M-9Br]^9+^ 203.59118.


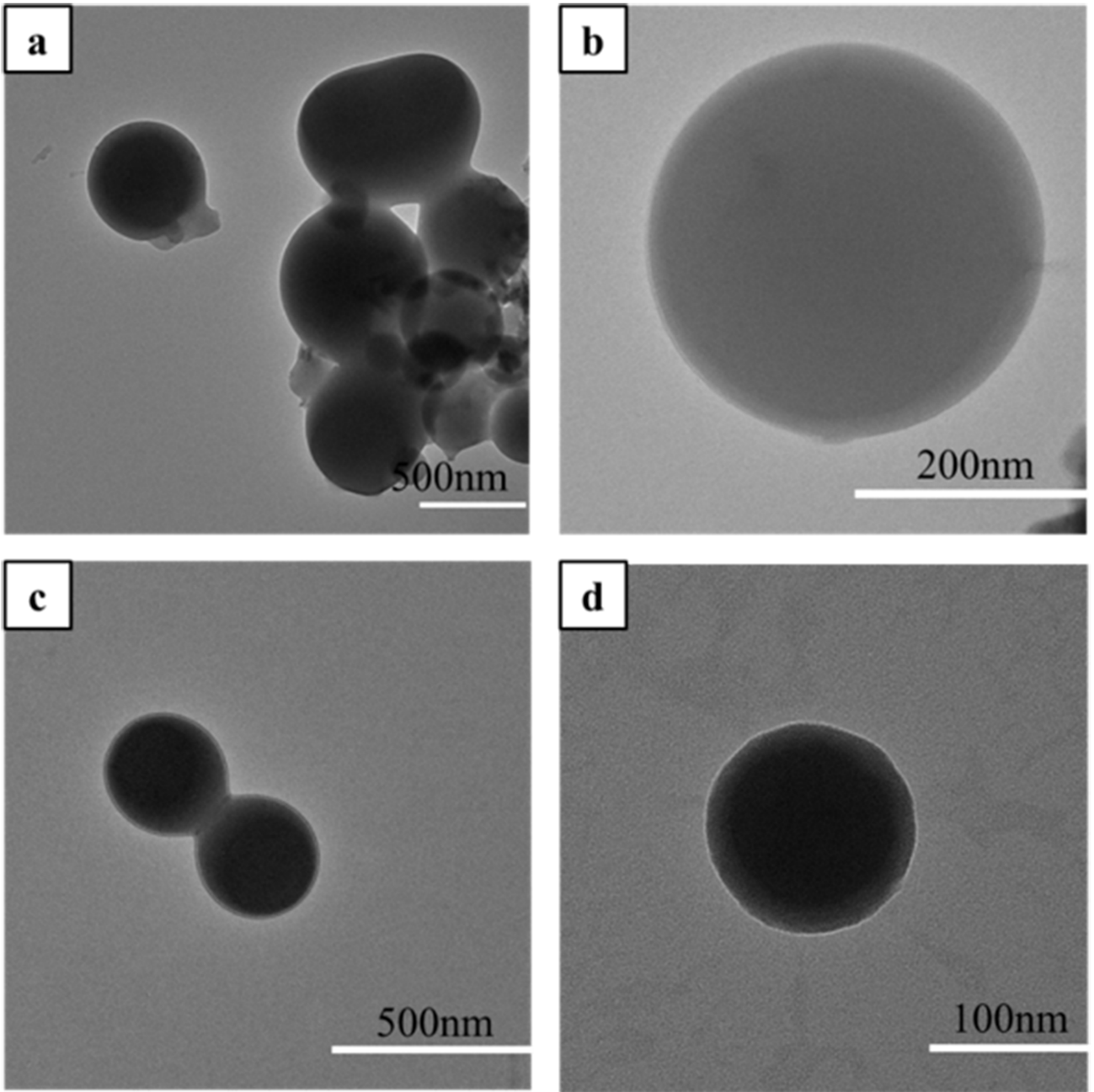


**Supplementary Figure 5.** TEM images of P5A-CrMo_6_ water solution with charge ratios at 4:10 (a); 6:10 (b); 8:10 (c) and 10:10 (d) at a concentration of 0.05 mg / mL. For charge ratios at 4:10, 6:10, 8:10, 10:10, the hydrodynamic radius are 567 nm, 295 nm, 130 nm and 75 nm, respectively.


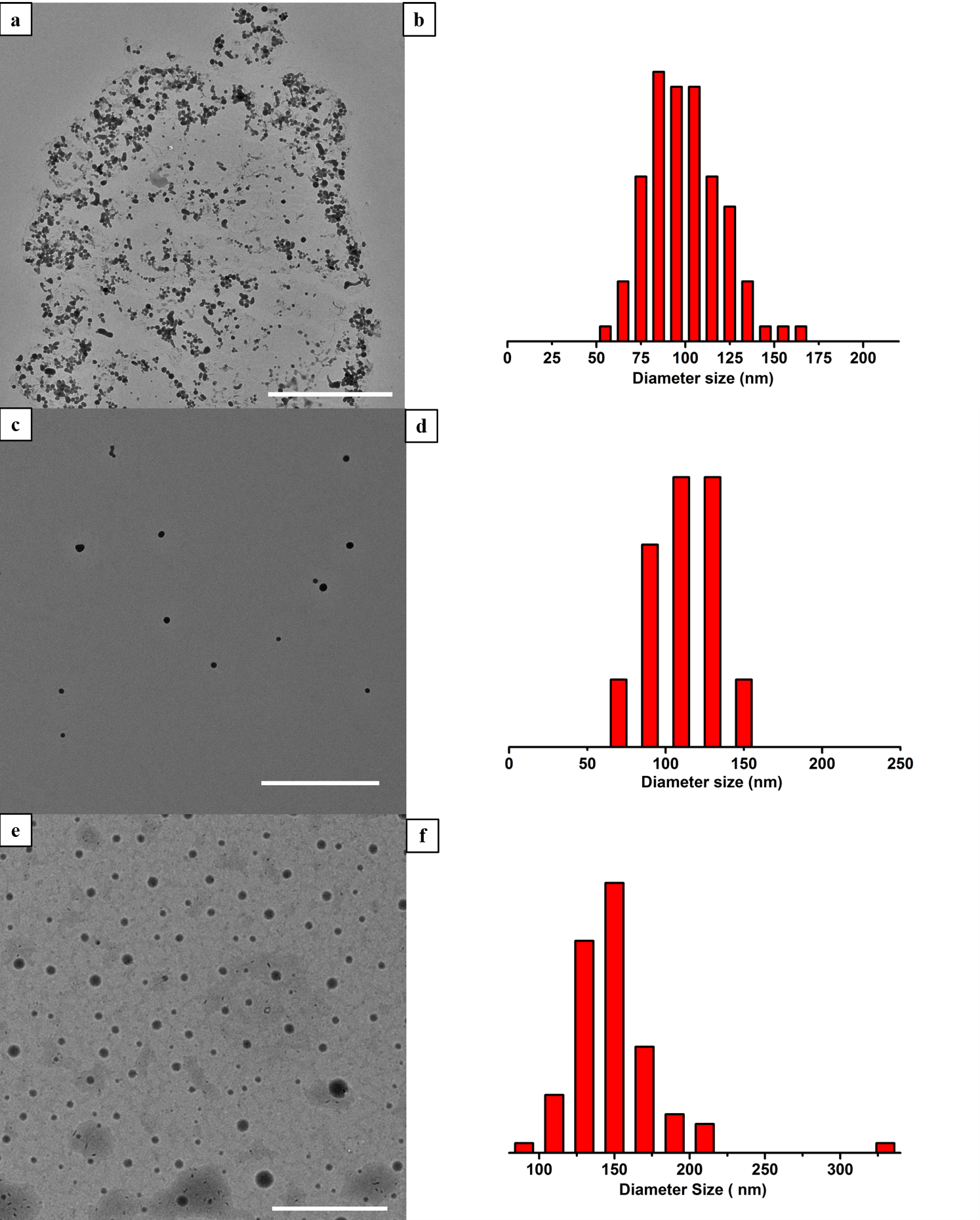


**Supplementary Figure 6.** TEM images of nanospheres formed by P5A-CrMo_6_ in water solution with charge ratios of 10:10 at 0.05 mg/mL (a) at the 1^st^ day; (b)the size-dispersion histogram of nanosphere at the 1^st^ day; (c) at the 8th day; (d)the size-dispersion histogram of nanosphere at the 8^th^ day; (e) at the 17^th^ day; (f)the size-dispersion histogram of nanosphere at the 17^th^ day. The scale bar : 2 μm.


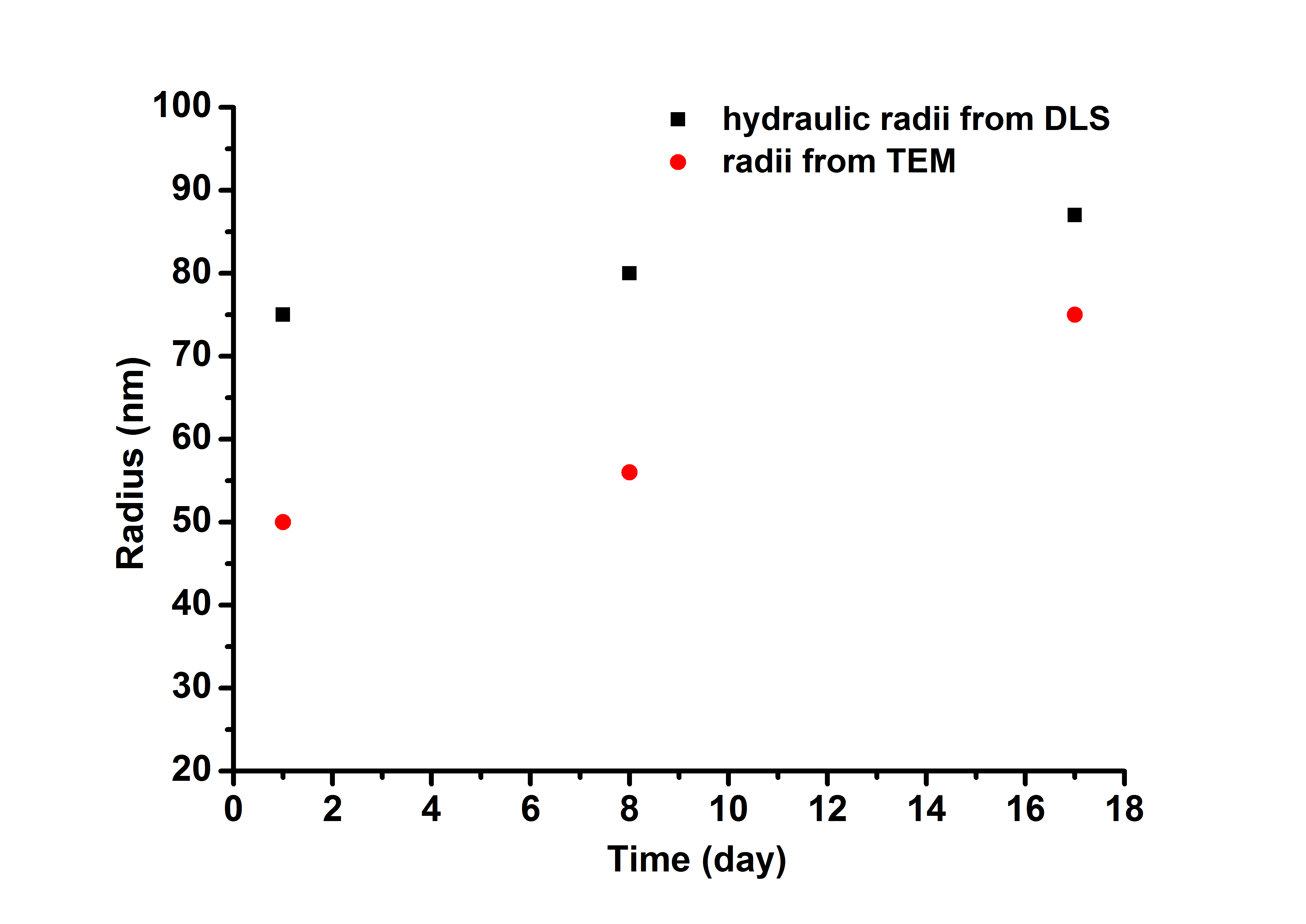


**Supplementary Figure 7.** The correlation between hydrodynamic radii of nanospheres from DLS and the radii of nanospheres from TEM.


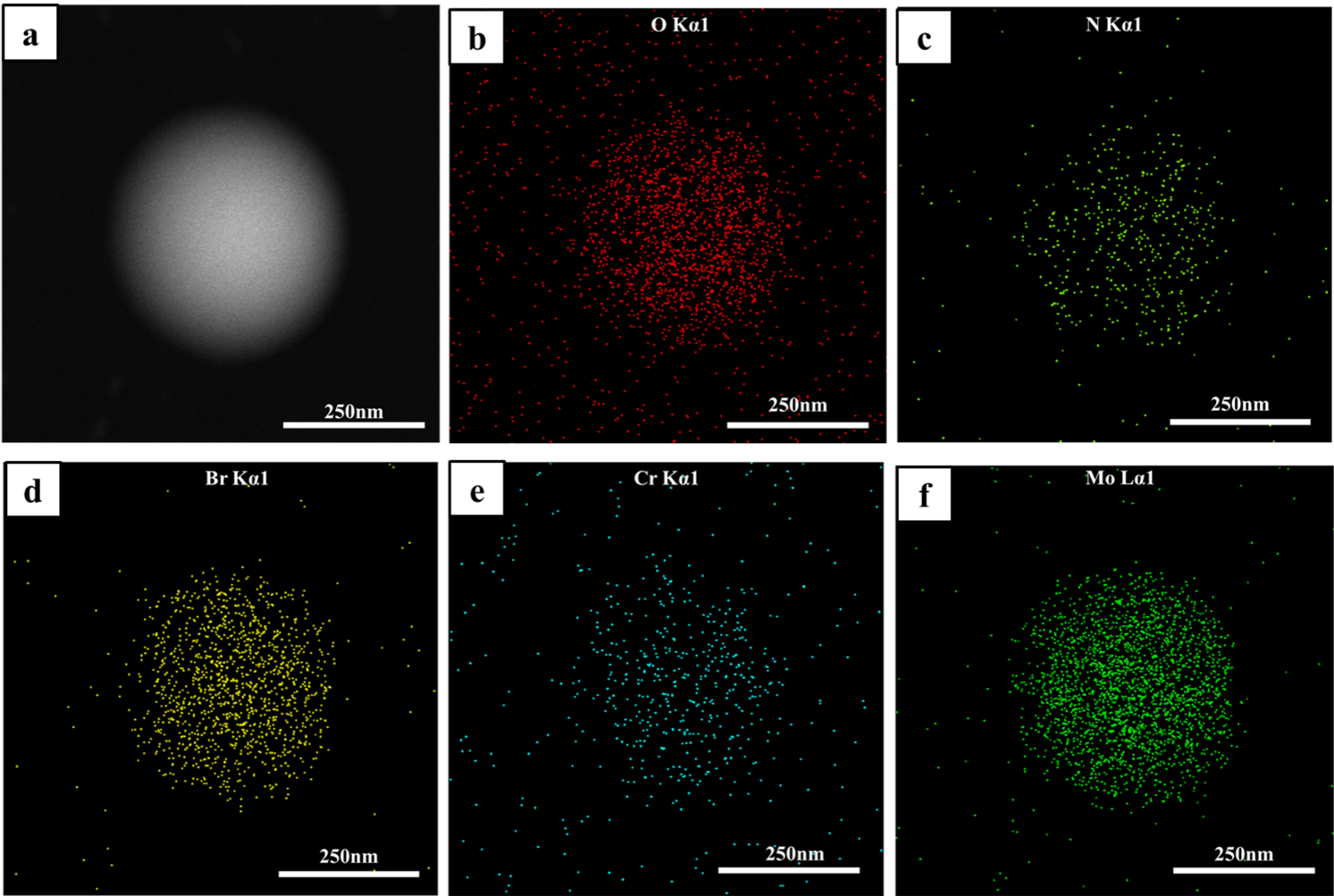


**Supplementary Figure 8.** EDX mapping of (b) O, (c) N, (d) Br, (e) Cr and (f) Na. Scale bar: 100 nm.

**Supplementary Table 1.** The elemental analysis of complex P5A-CrMo_6_.

| **Sample Name** | **(N)%** | **(C)%** | **(H)%** | **(O)%** |
| --- | --- | --- | --- | --- |
| **P5A-CrMo_6_** | **2.695** | **24.355** | **4.387** | **27.739** |


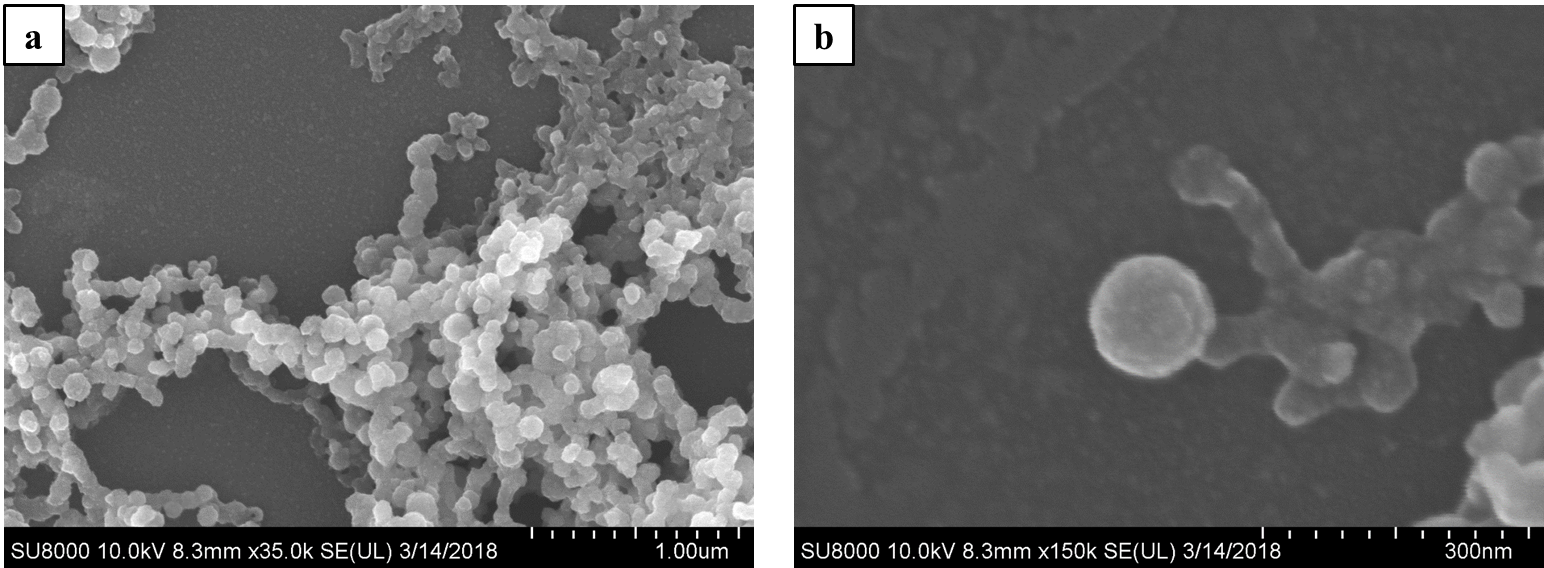


**Supplementary Figure 9.** SEM images of nanospheres formed by P5A-CrMo_6_ in water solution with charge ratios of 10:10 at 0.05 mg/mL (a) and the size-dispersion histogram of nanospheres (b).


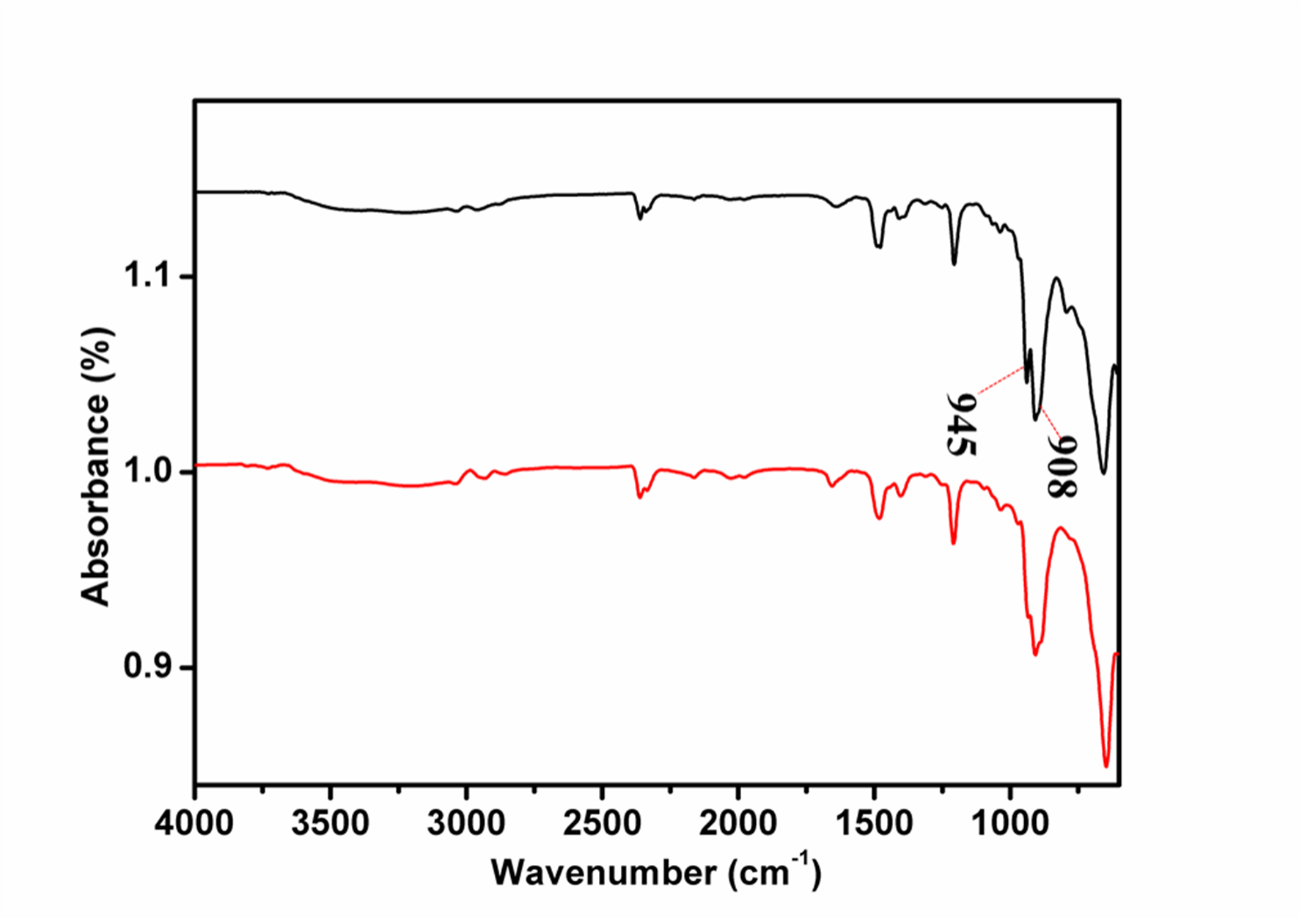


**Supplementary Figure 10.** IR spectrum of P5A-CrMo_6_ (black line) and after six catalytic cycles (red line).


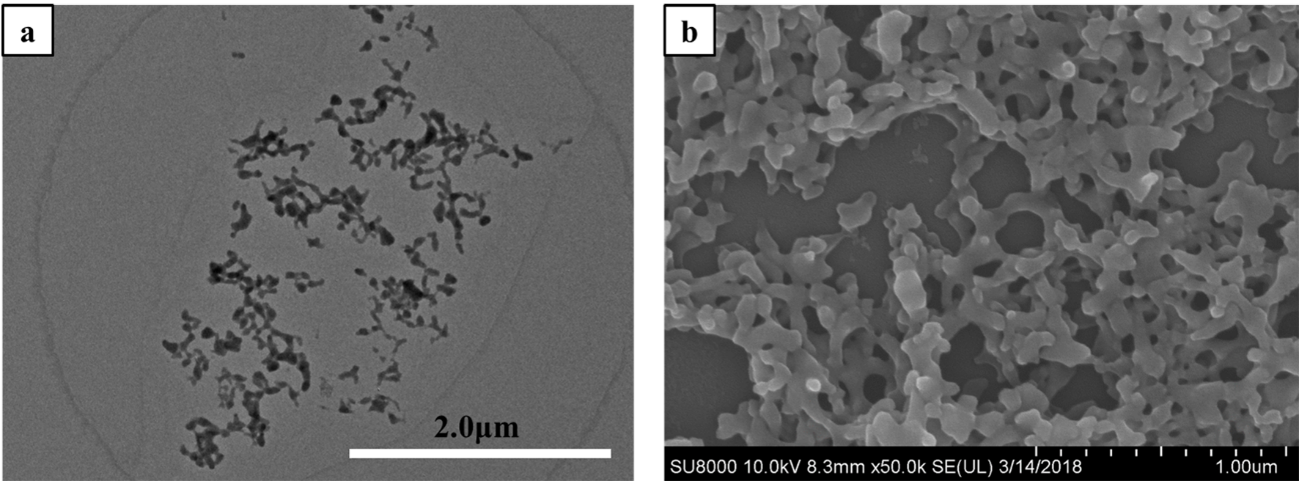


**Supplementary Figure 11.** TEM images (a) and SEM images (b) of P5A-CrMo_6_ after catalytic reaction.

All compounds are previously known, and the data reported herein are consistent with the literature reports (Liu et al., 2015; Liu and Li, 2016; Yu et al., 2017; Zhang et al., 2017).

**Benzoic acid:** ^1^H- NMR (300 MHz, DMSO) δ 12.96 (s, 1H), 7.96 (d, *J* = 7.2 Hz, 2H), 7.62 (t, *J* = 7.4 Hz, 1H), 7.50 (t, *J* = 7.7 Hz, 2H); ^13^C-NMR (300 MHz, DMSO) δ 167.79 (s), 133.30 (s), 131.24 (s), 129.73 (s), 129.01 (s).

**4-Methylbenzoic acid:** ^1^H-NMR (300 MHz, DMSO) δ 12.78 (s, 1H), 7.84 (d, *J* = 8.1 Hz, 2H), 7.30 (d, *J* = 7.9 Hz, 2H), 2.37 (s, 3H);^13^C-NMR (300 MHz, DMSO) δ167.77 (s), 143.48 (s), 129.72 (d, *J* = 18.9 Hz), 21.58 (s).

**4-Bromobenzoic acid:** ^1^H-NMR (300 MHz, DMSO) δ 13.18 (s, 1H), 7.94 (d, *J* = 8.5 Hz, 2H), 7.57 (d, *J* = 8.4 Hz, 2H); ^13^C-NMR (300 MHz, DMSO) δ 166.53 (s), 131.68–131.29 (m), 130.04 (s), 126.87 (s).

**4-Nitrobenzoic acid:** ^1^H-NMR (300 MHz, DMSO) δ 13.67 (s, 1H), 8.33 (d, *J* = 8.8 Hz, 2H), 8.17 (d, *J* = 8.8 Hz, 2H); ^13^C-NMR (300 MHz, DMSO) δ 166.26 (s),150.50 (s), 136.85 (s), 131.15 (s), 124.17 (s).

**4-Hydroxybenzoic acid:** ^1^H-NMR (300 MHz, DMSO) δ 12.41 (s, 1H), 10.20 (s, 1H), 7.79 (d, *J* = 8.6 Hz, 2H), 6.82 (d, *J* = 8.6 Hz, 2H); ^13^C-NMR (300 MHz, DMSO) δ 167.62 (s), 162.06 (s), 131.98 (s), 121.85 (s), 115.58 (s).

**4-Methoxybenzoic acid:** ^1^H-NMR (300 MHz, DMSO) δ 12.61 (s, 1H), 7.88 (d, *J* = 8.6 Hz, 2H), 6.99 (d, *J* = 8.6 Hz, 2H), 3.81(s, 3H); ^13^C-NMR (300 MHz, DMSO) δ 167.05 (s), 162.87 (s), 131.38 (s), 123.01 (s), 113.83 (s).

**Salicylic acid:** ^1^H-NMR (300 MHz, DMSO) δ 7.80 (dd, *J* = 7.9, 1.5 Hz, 1H),7.56 – 7.47 (m, 1H), 6.94 (dd, *J* = 17.1, 7.9 Hz, 2H); ^13^C-NMR (300 MHz, DMSO) δ172.37 (s), 161.59 (s), 136.12 (s), 130.74 (s), 119.59 (s), 117.56 – 117.40 (m), 113.32 (s).

**Nicotnic acid:** ^1^H-NMR (300 MHz, DMSO) δ 13.43 (s, 1H), 9.08 (d, *J* =1.2 Hz, 1H), 8.79 (dd, *J* = 4.6, 1.2 Hz, 1H), 8.27 (d, *J* = 7.9 Hz, 1H), 7.55 (dd, *J* = 7.8, 4.9 Hz, 1H). ^13^C-NMR (300 MHz, DMSO) δ 166.73 (s), 153.77 (s), 150.69 (s), 137.32 (s), 127.03 (s), 124.19 (s).

**Enanthic acid:**^1^H-NMR (300 MHz, DMSO) δ 11.92 (s, 1H), 2.17 (t, *J* = 7.4 Hz, 2H), 1.48 (dd, *J* = 14.3, 7.1 Hz, 2H), 1.26 (dd, *J* = 16.7, 4.4 Hz, 6H), 0.85 (t, *J* =6.8 Hz, 3H); ^13^C-NMR (300 MHz, DMSO) δ 174.82 (s), 34.09 (s), 31.47 (s), 28.73 (s), 24.92 (s), 22.44 (s), 14.19 (s).


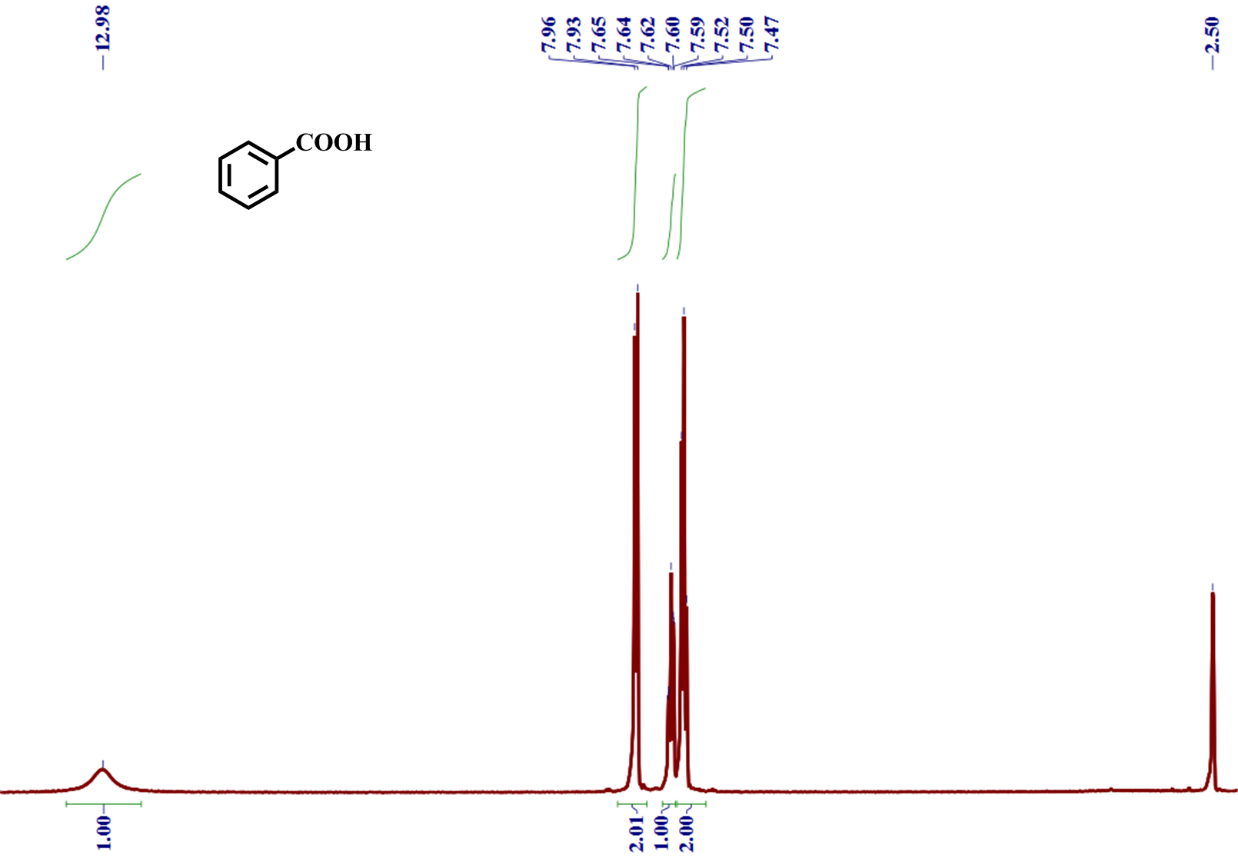


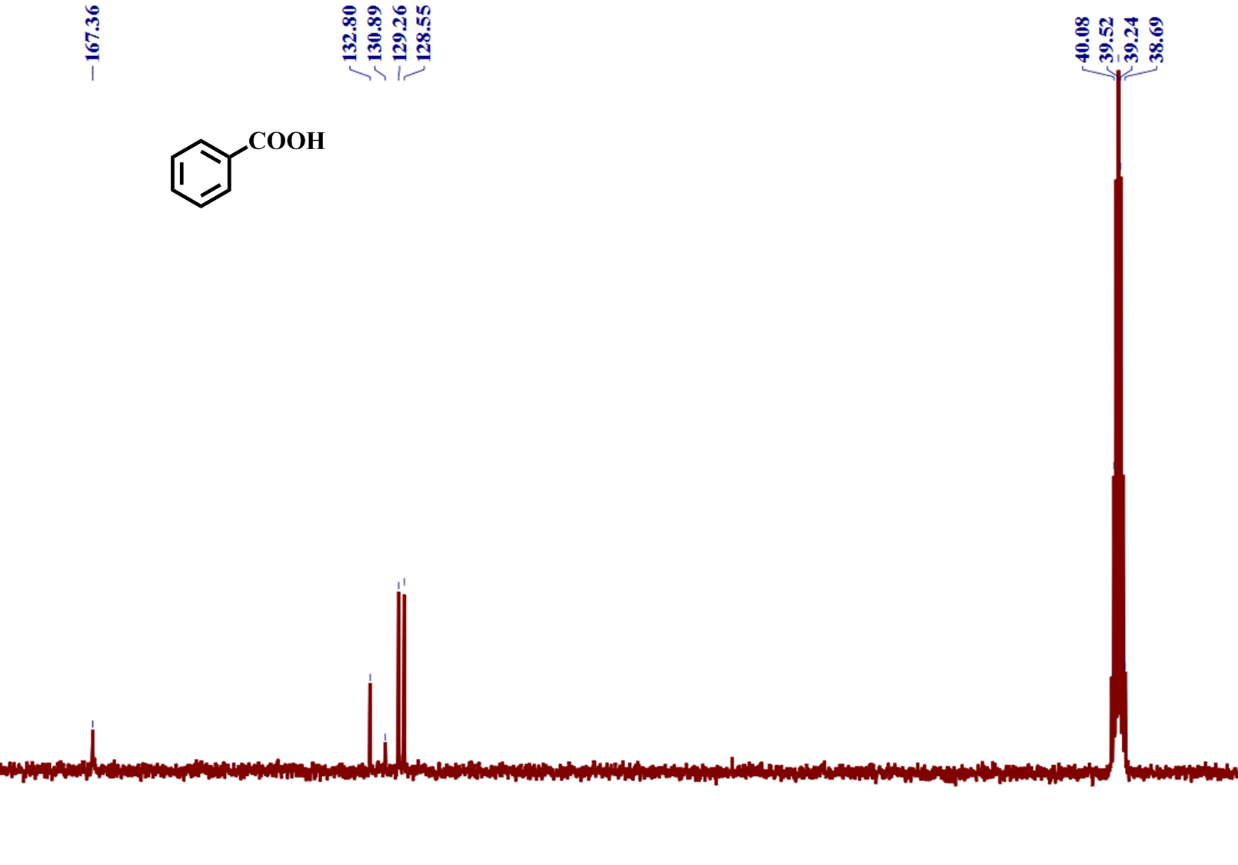


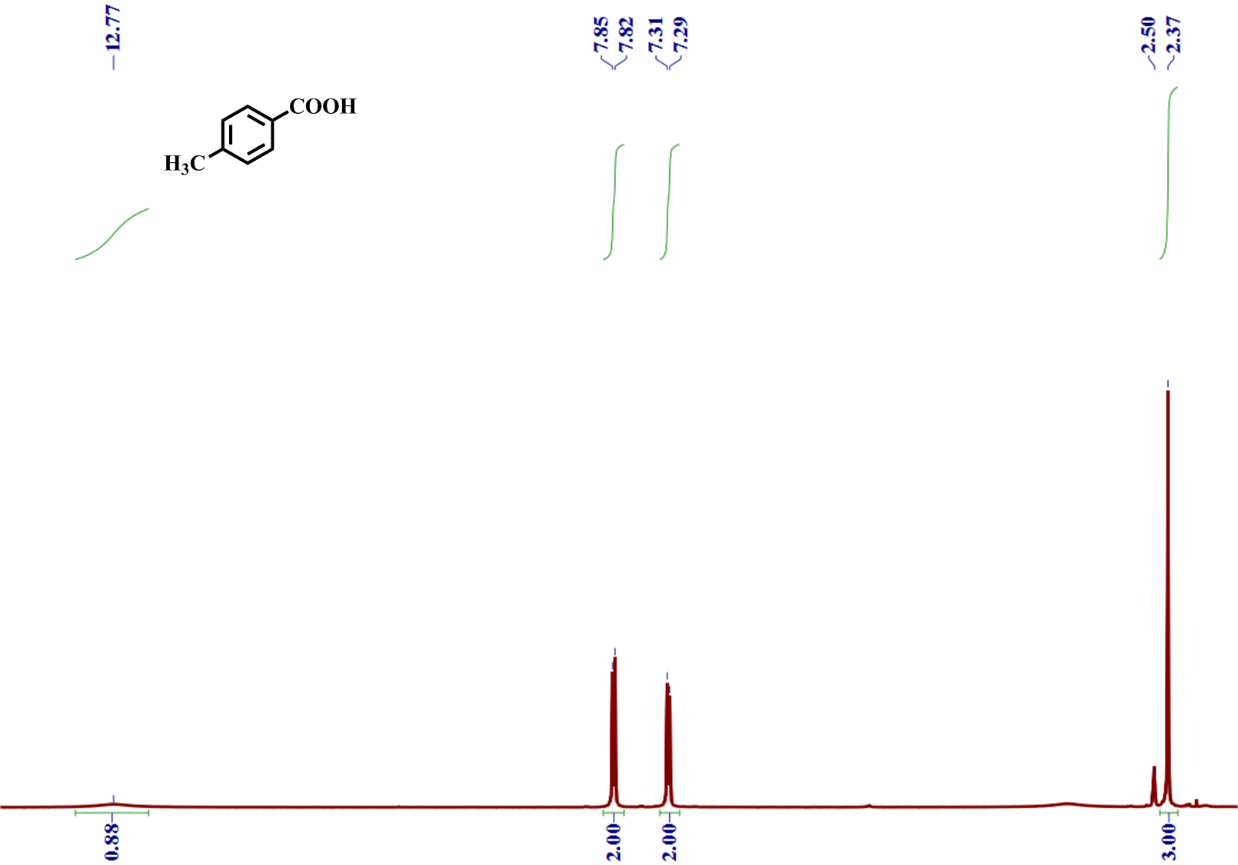


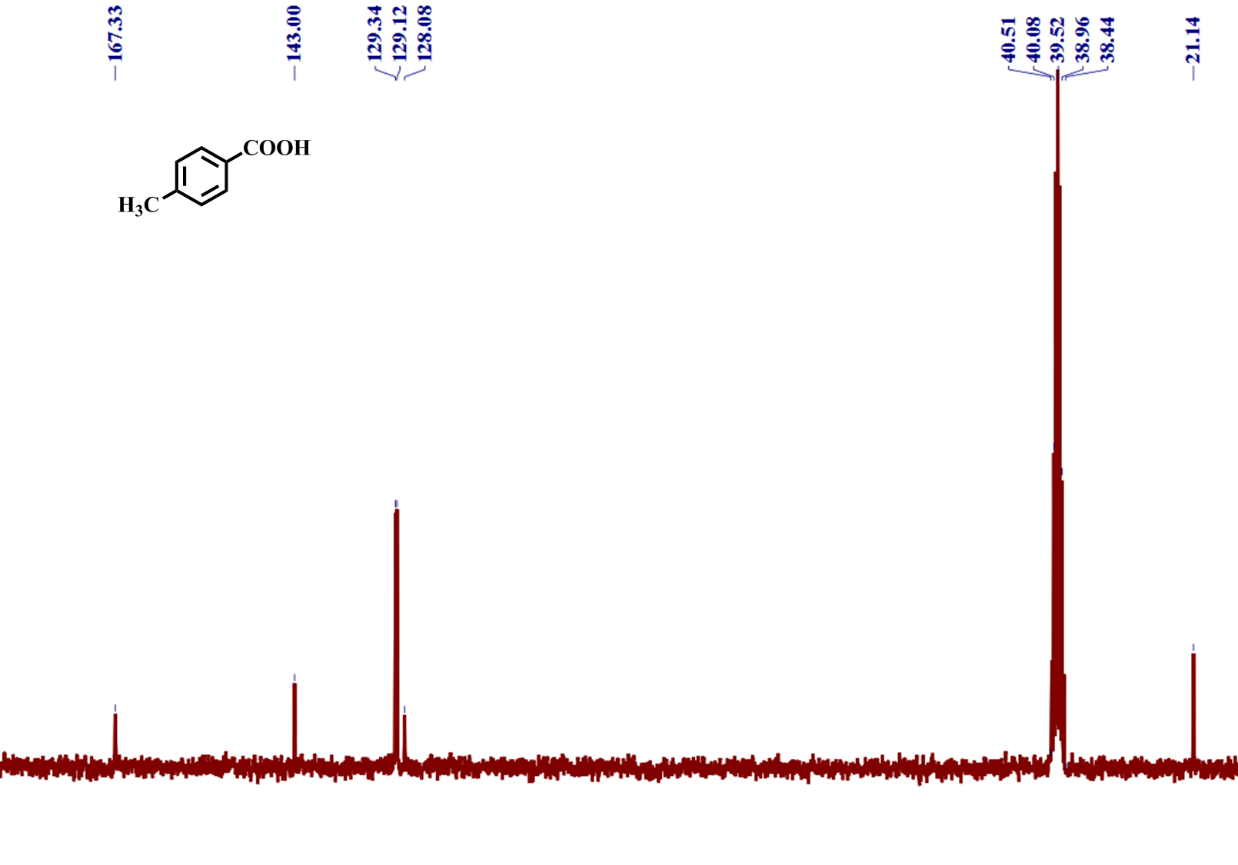


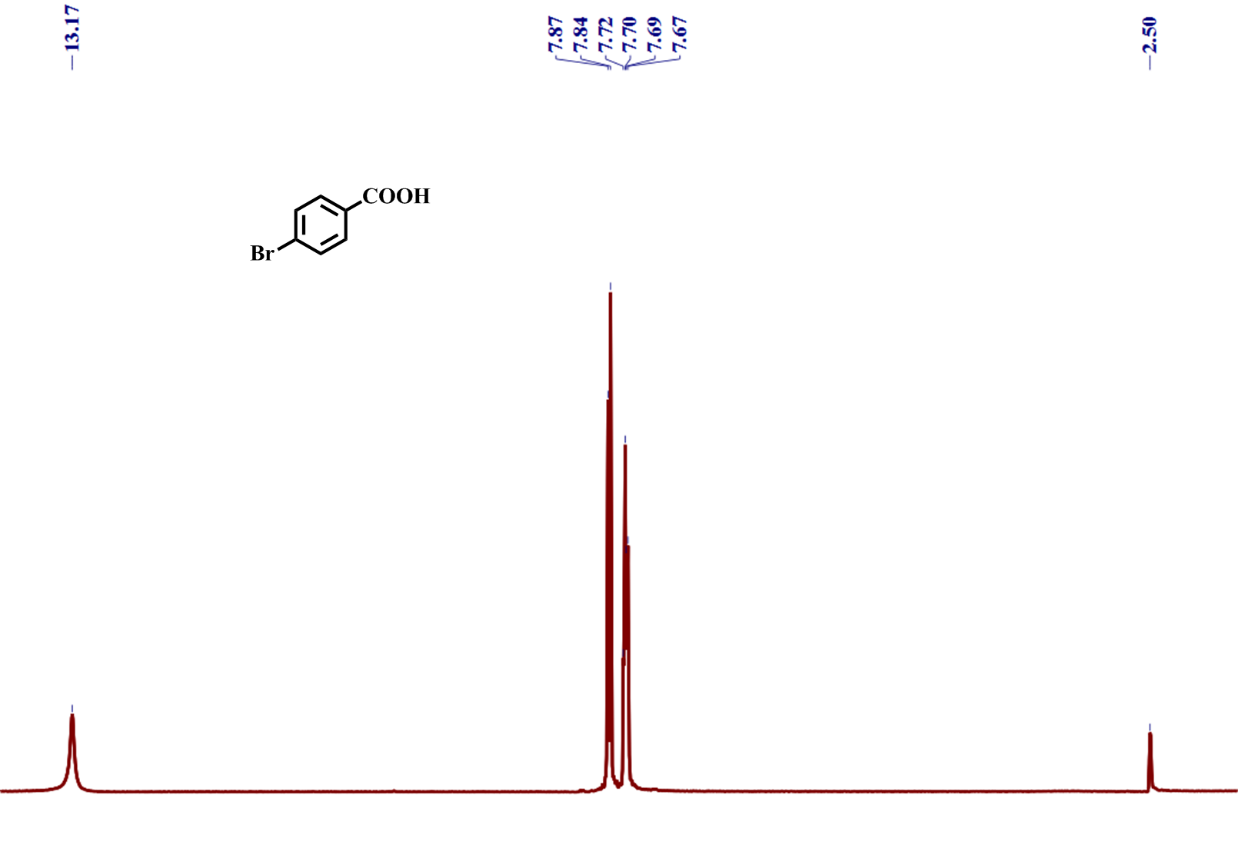


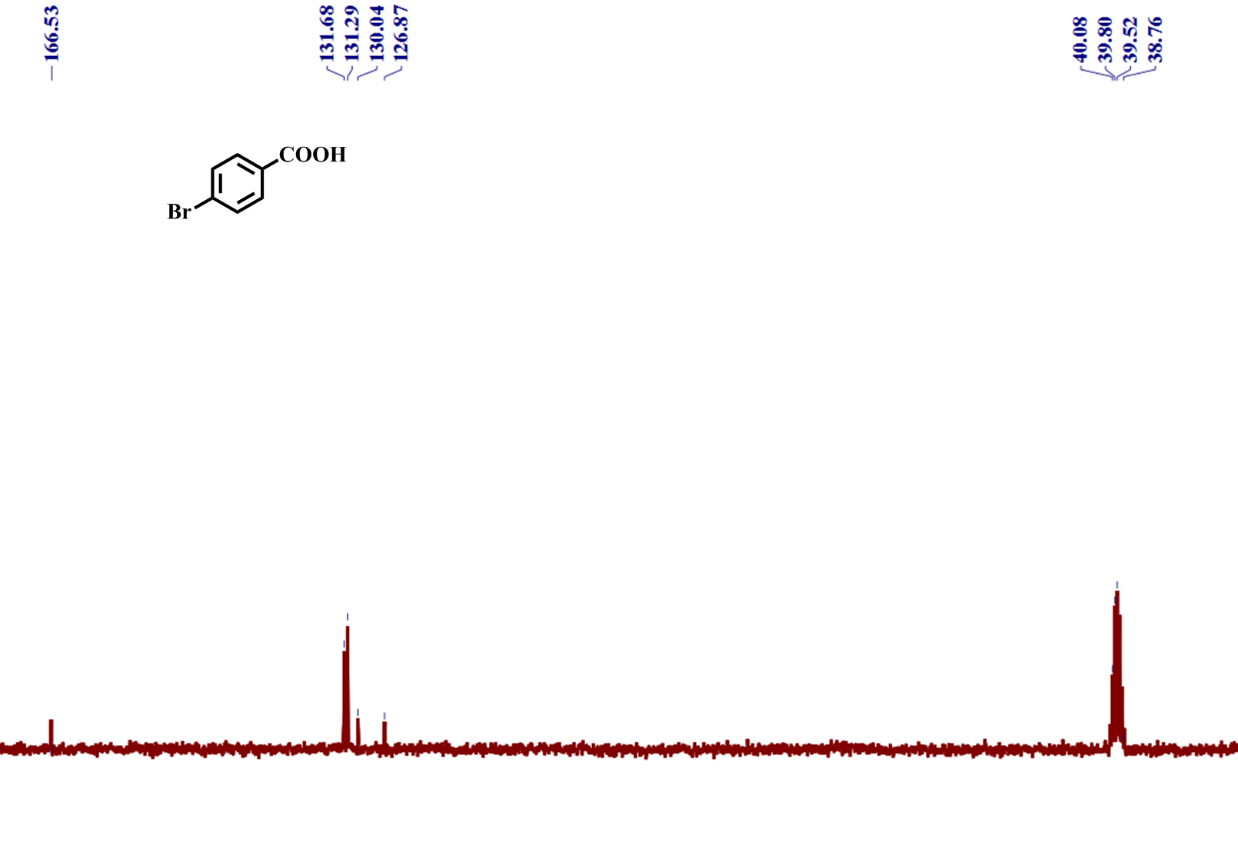


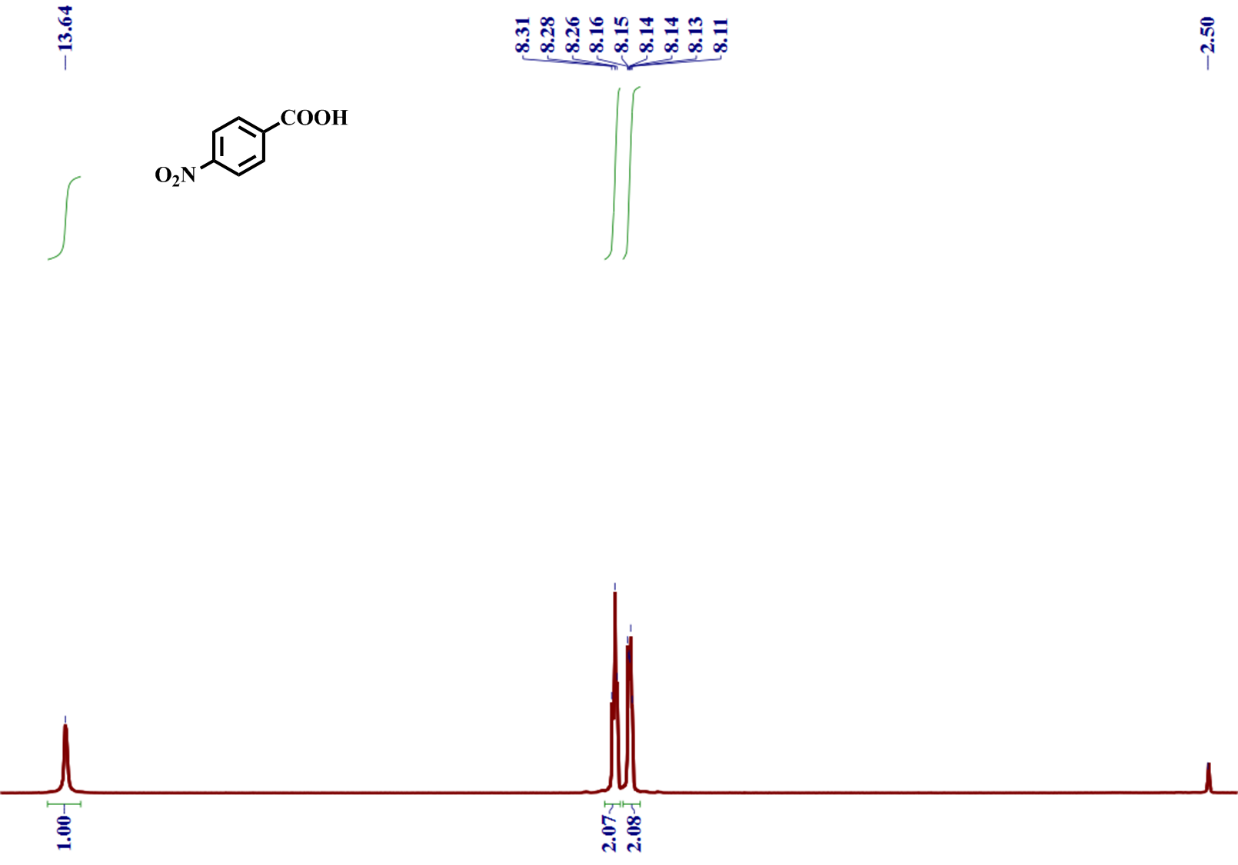


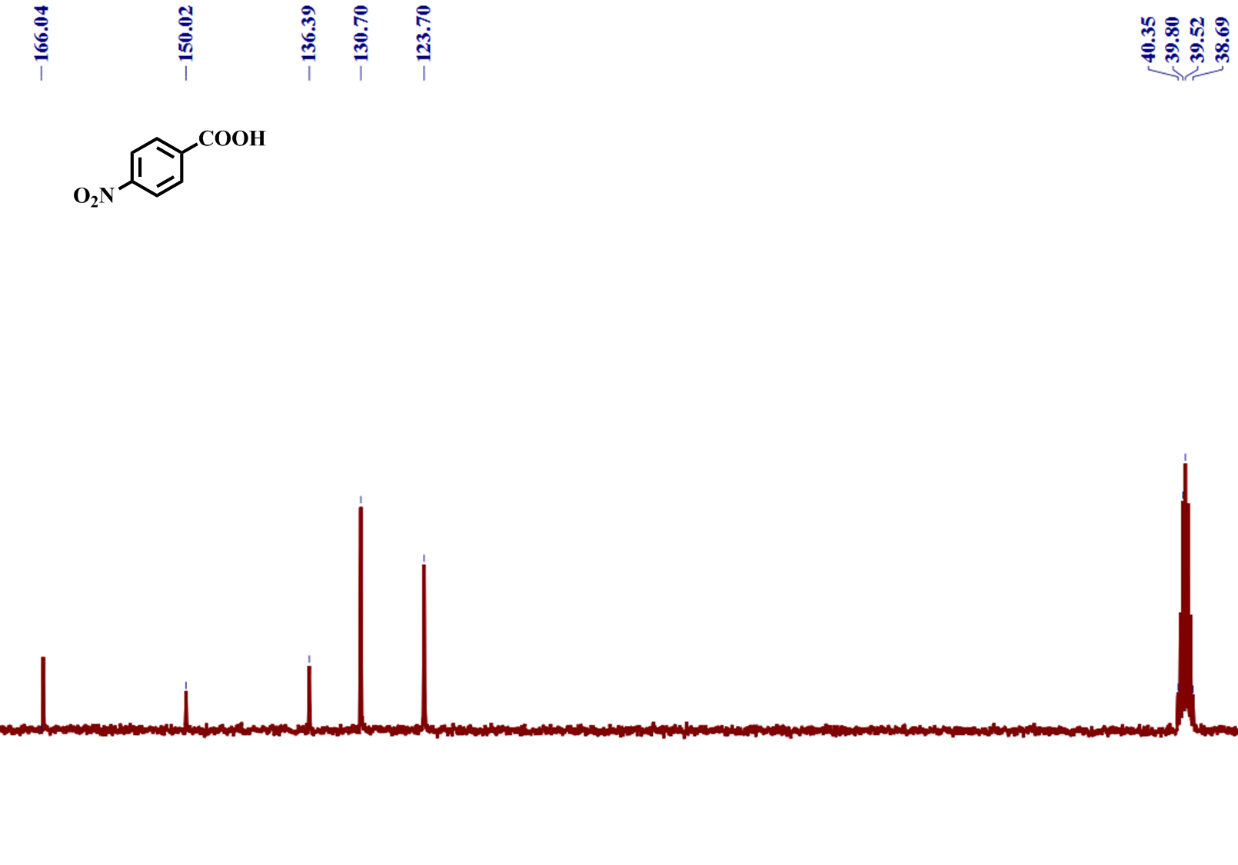


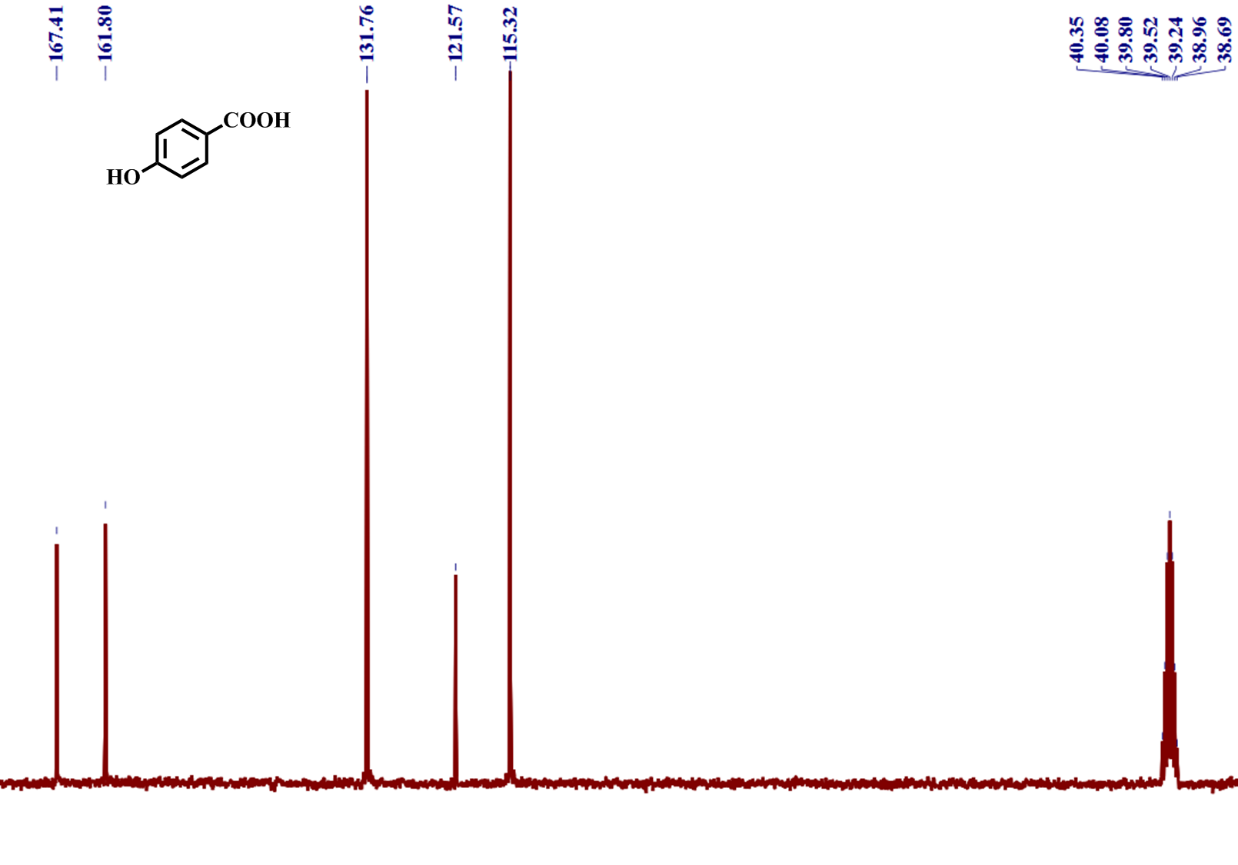


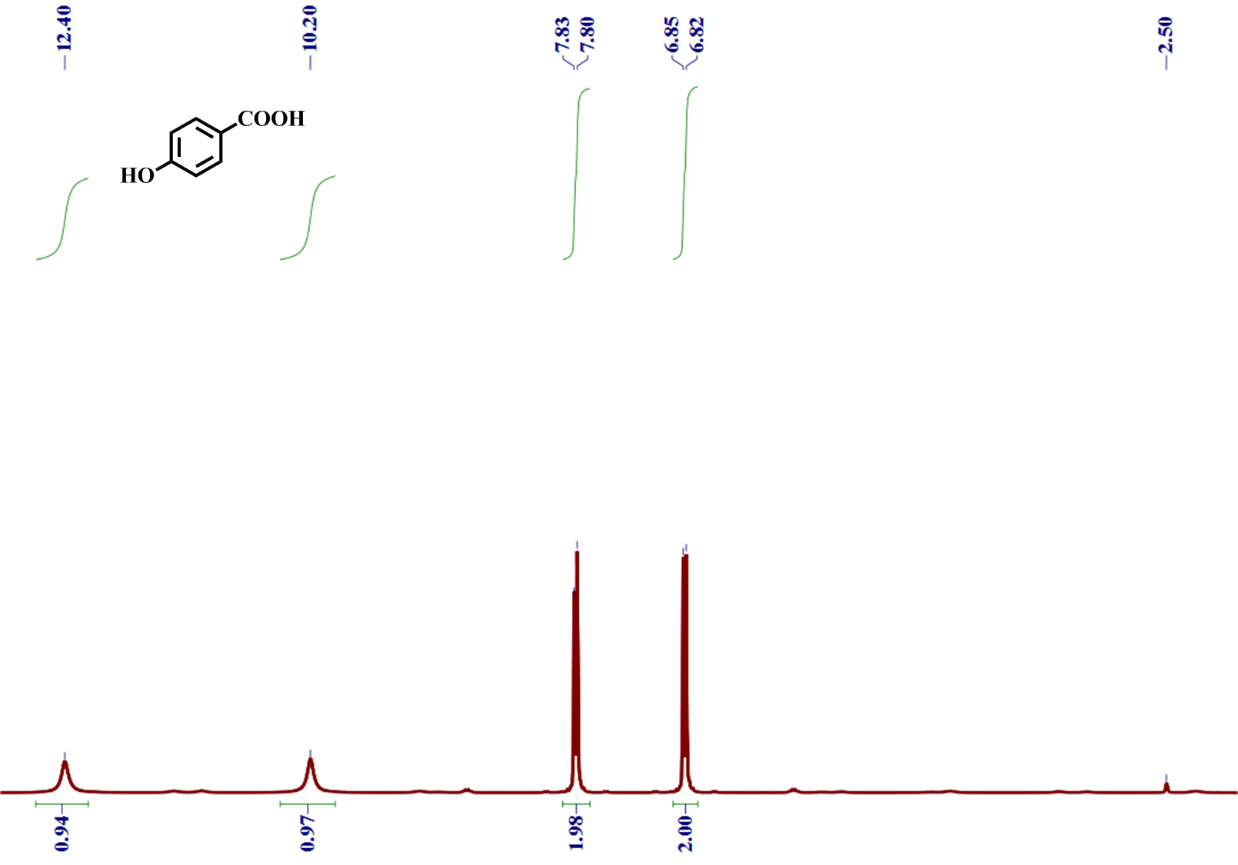


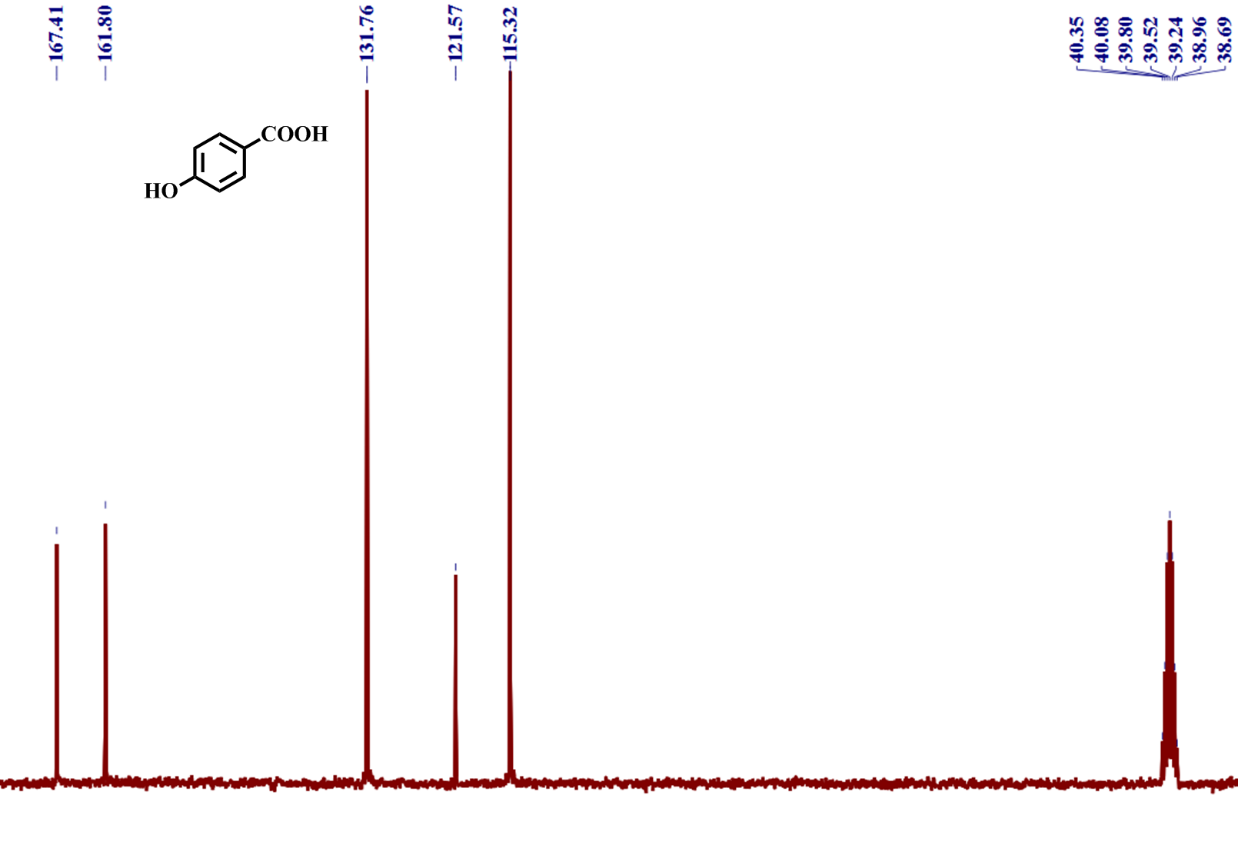


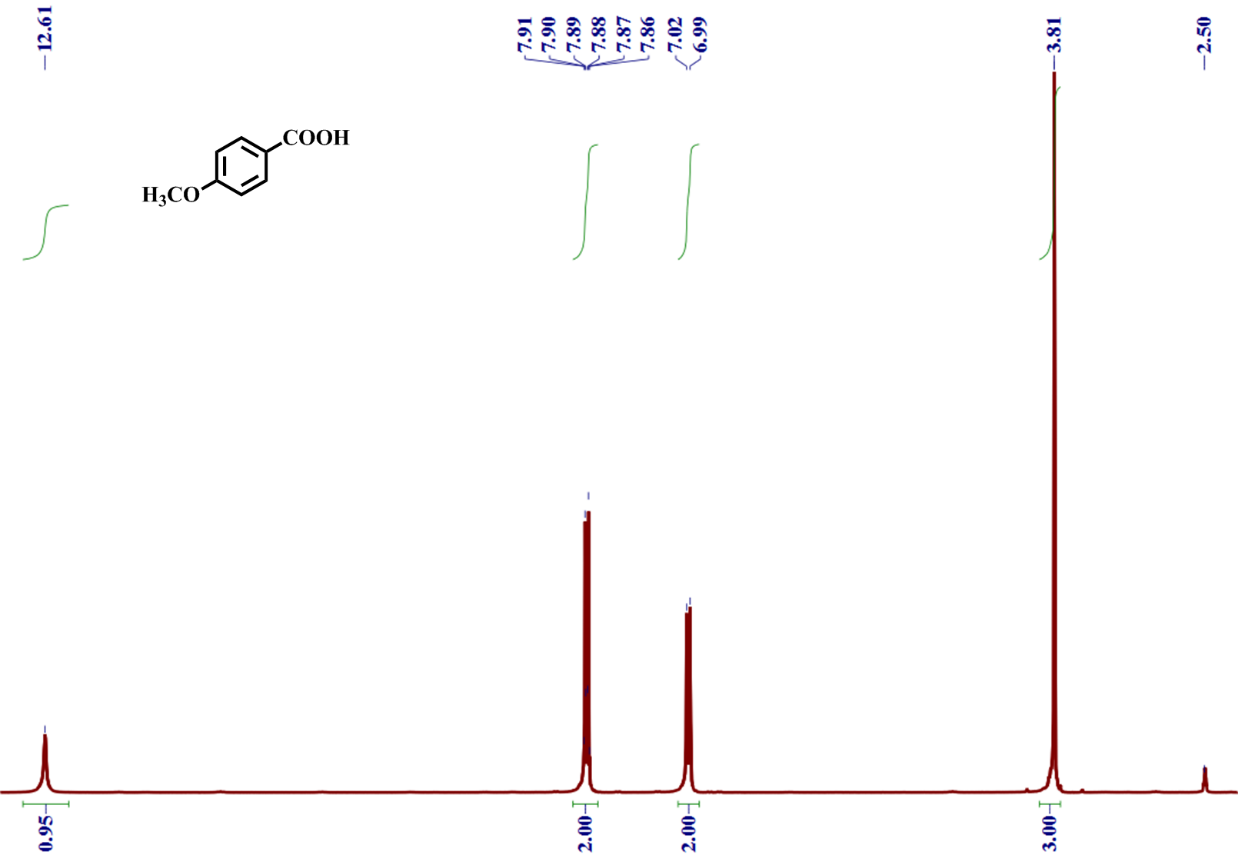


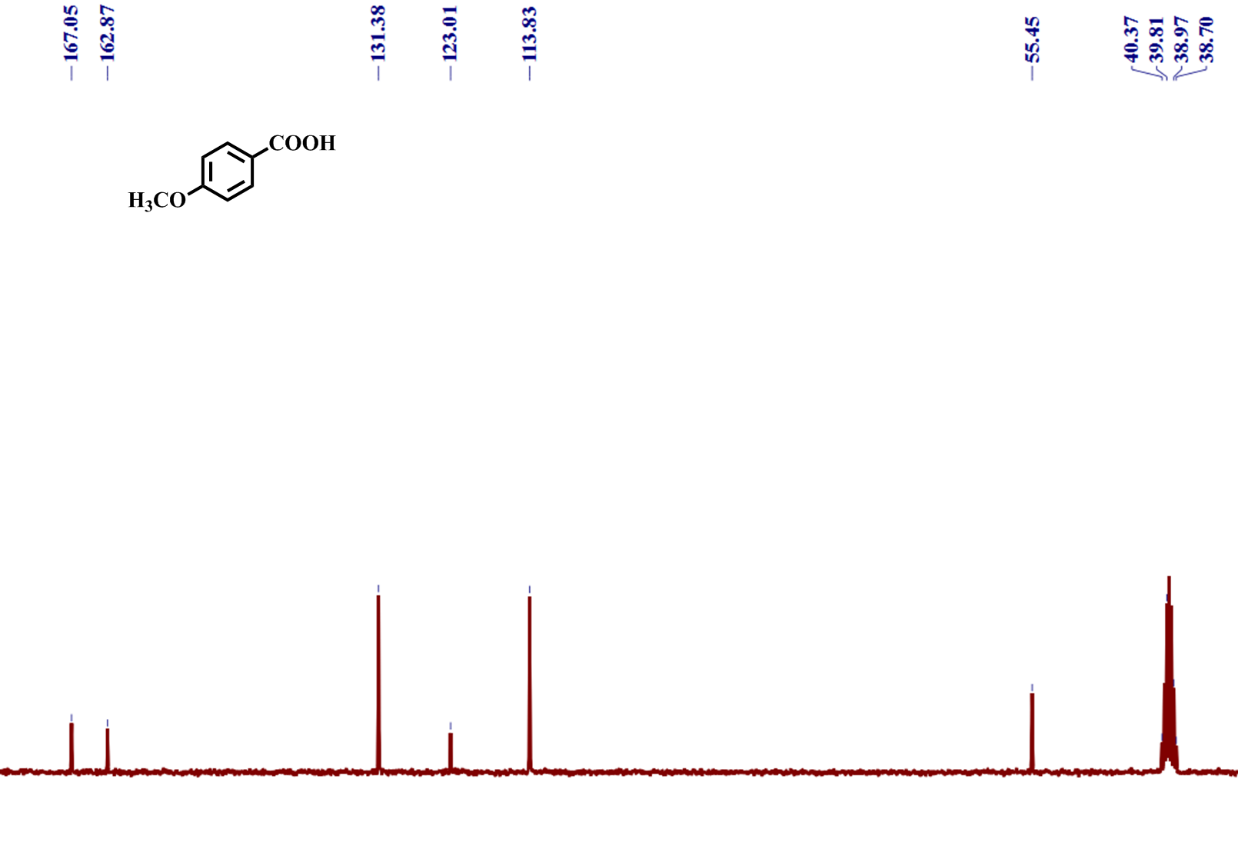


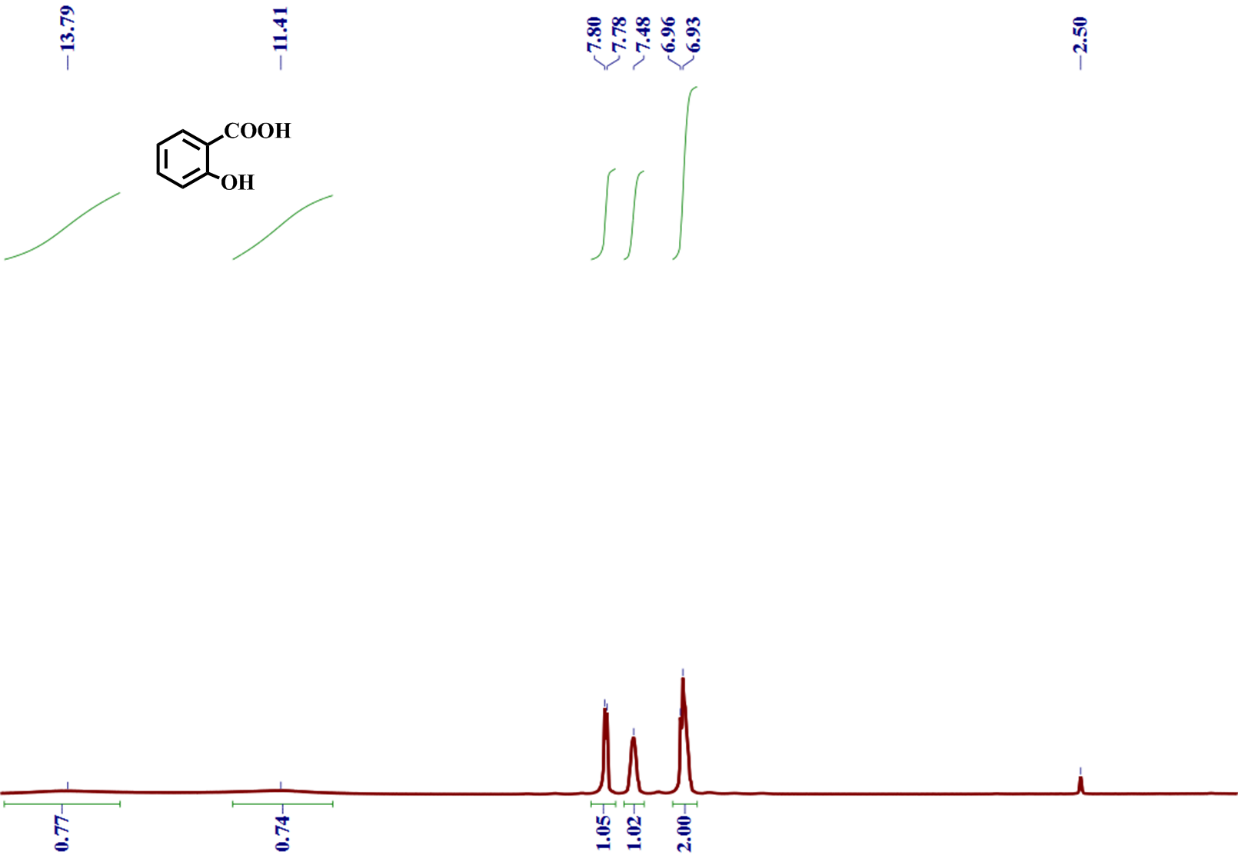


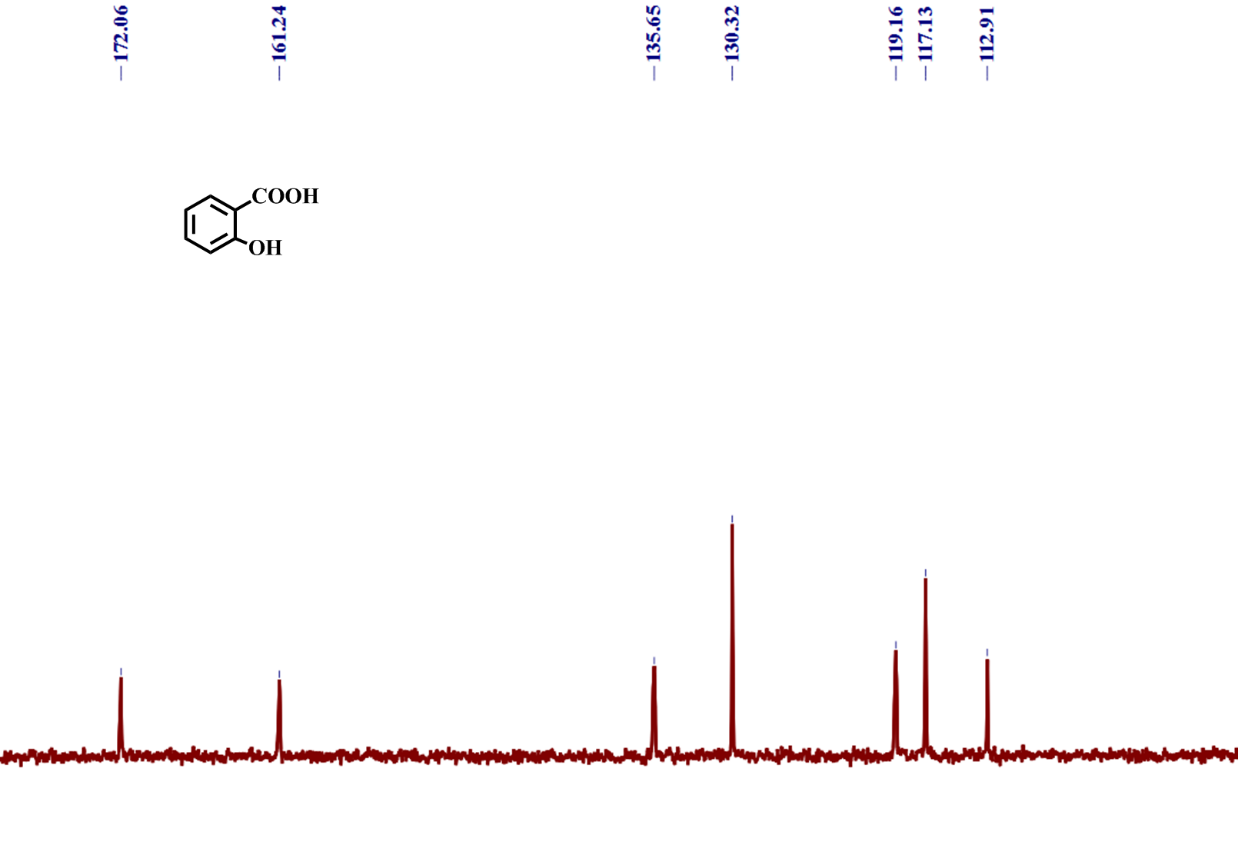


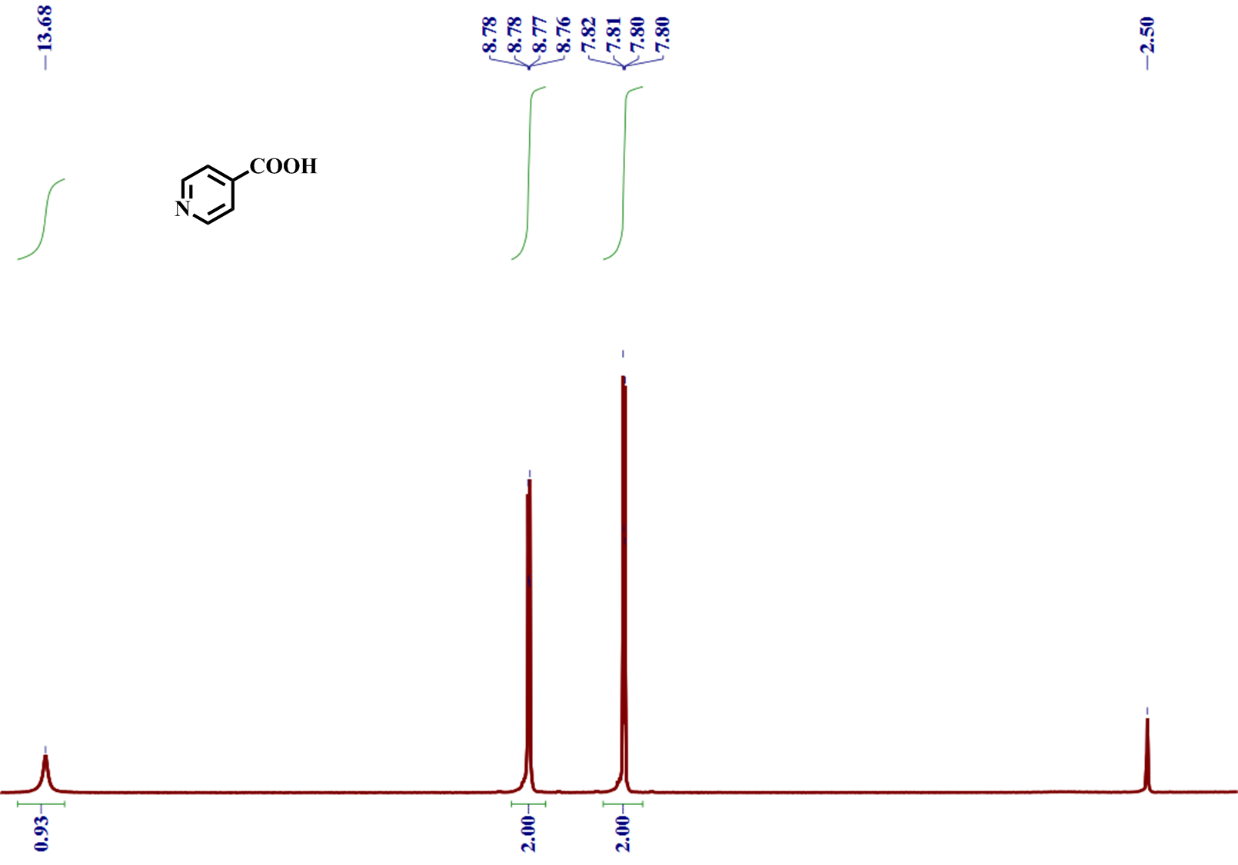


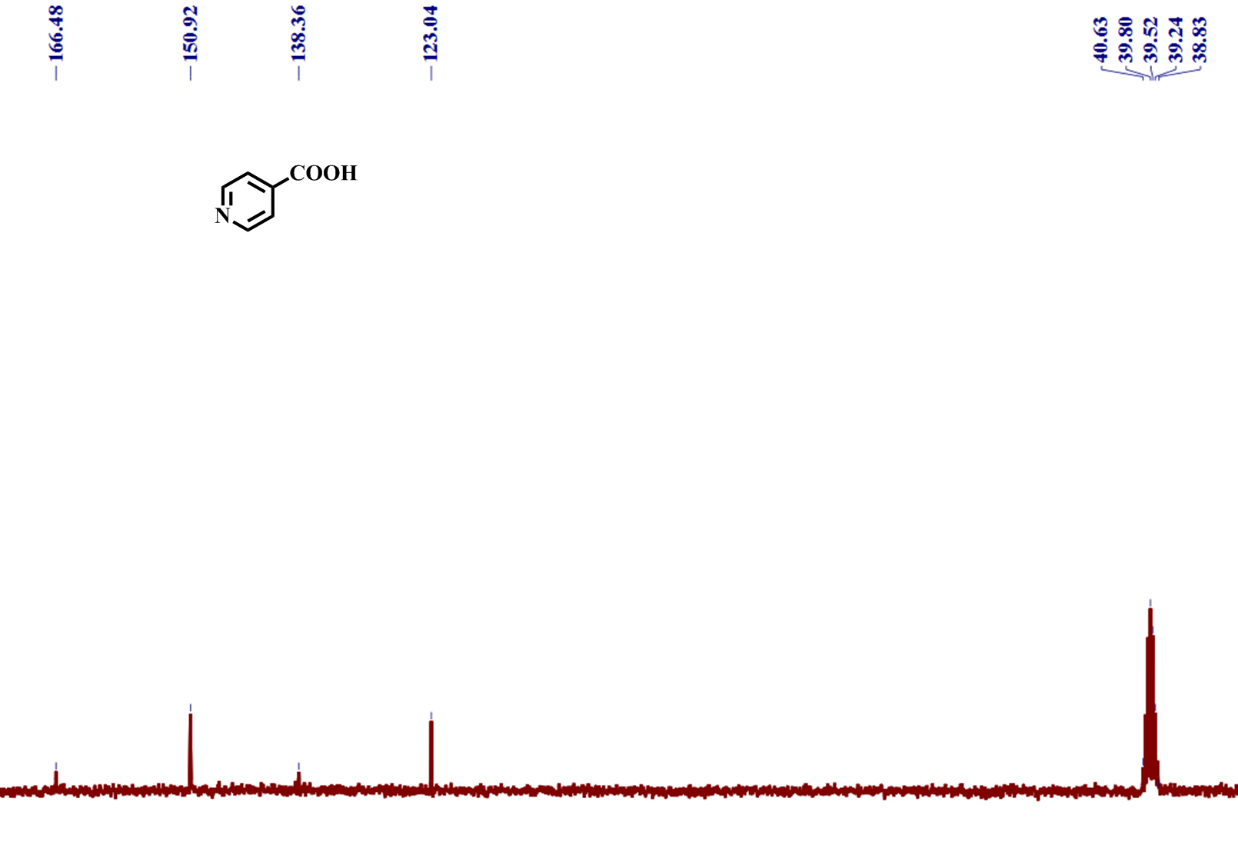


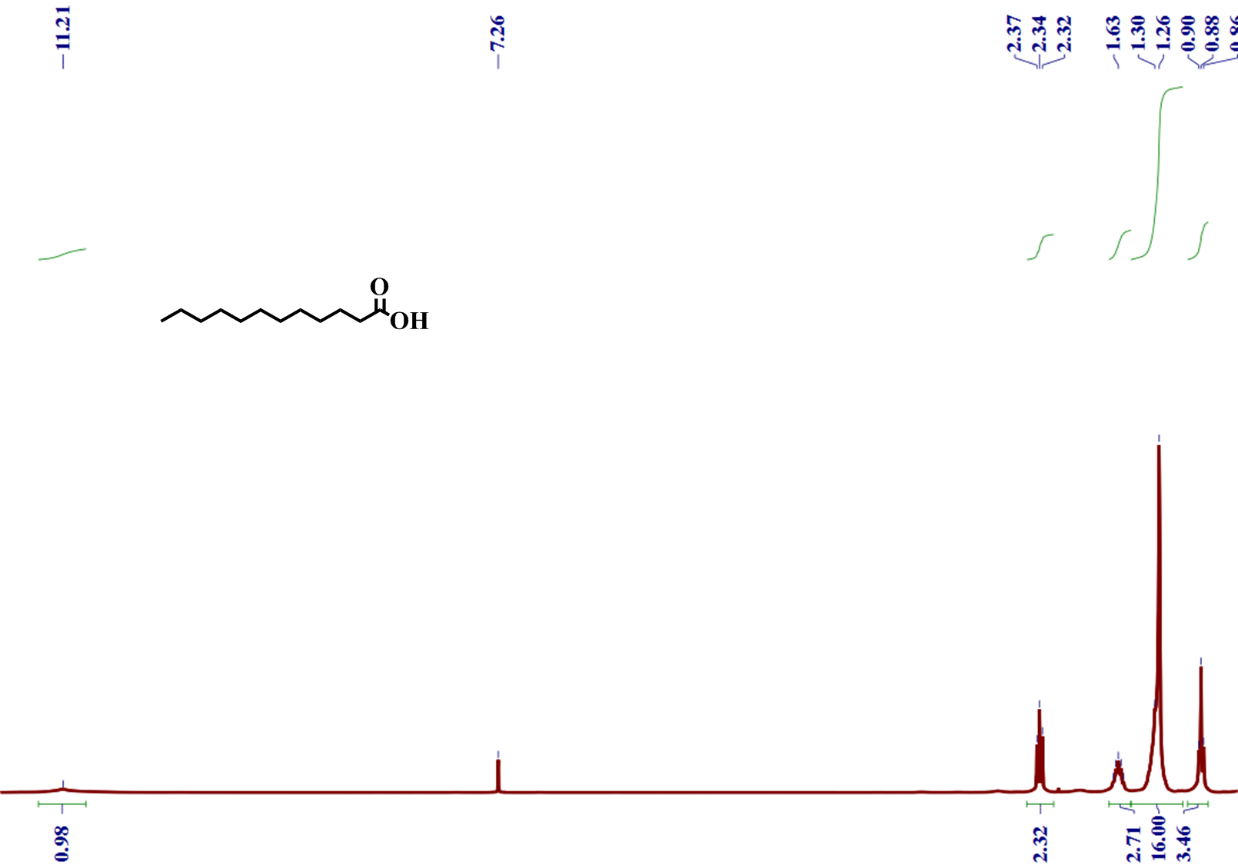


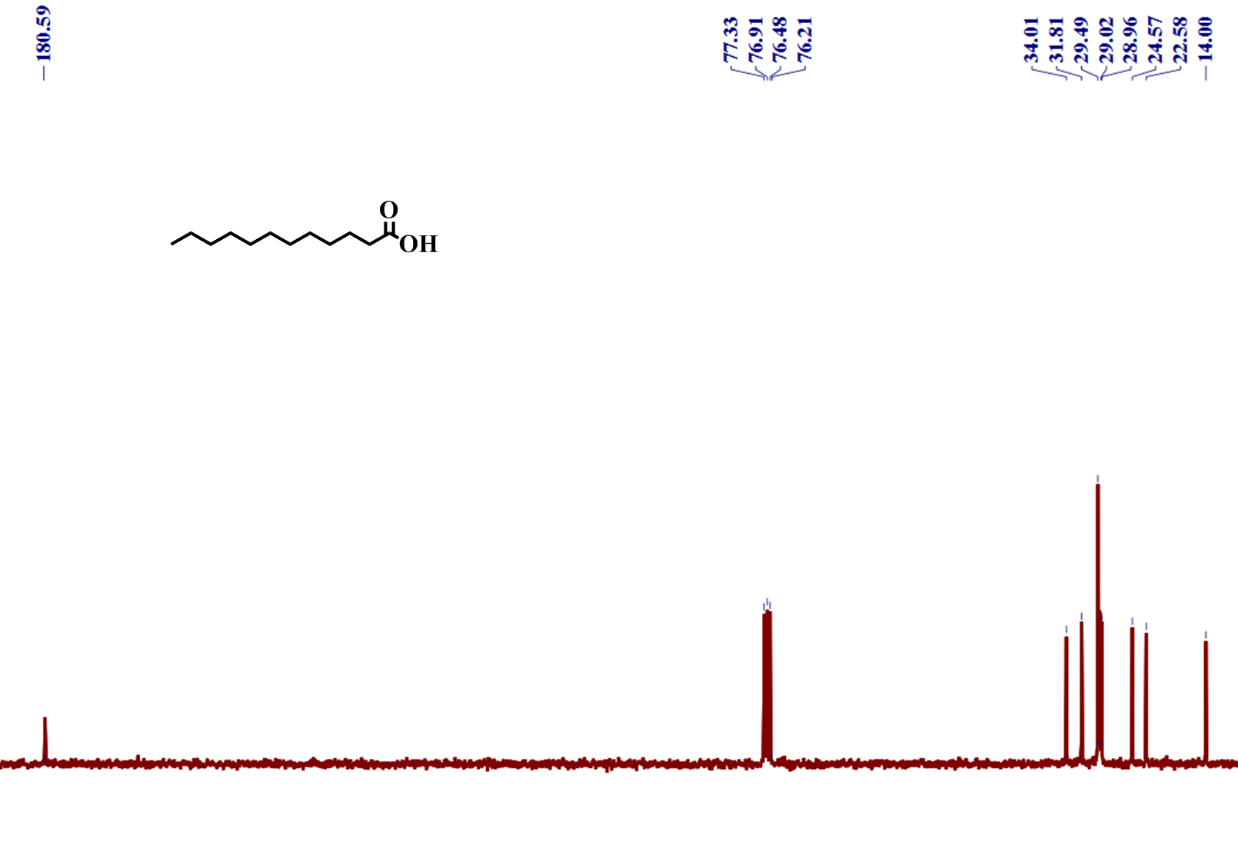


**Reference**

Liu, M., and Li, C.J. (2016). Catalytic fehling's reaction: an efficient aerobic oxidation of aldehyde catalyzed by copper in water. *Angew. Chem. Int. Ed.* 128(36)**,** 10964-10968. doi: 10.1002/anie.201604847.

Liu, M., Wang, H., Zeng, H., and Li, C.J. (2015). Silver(I) as a widely applicable, homogeneous catalyst for aerobic oxidation of aldehydes toward carboxylic acids in water-“silver mirror”: From stoichiometric to catalytic. *Sci. Adv.* 1(2)**,** 1500020-1500030. doi: 10.1126/sciadv.1500020

Yu, H., Ru, S., Dai, G., Zhai, Y., Lin, H., Han, S., et al. (2017). An efficient iron(III)-catalyzed aerobic oxidation of aldehydes in water for the green preparation of carboxylic acids. *Angew. Chem. Int. Ed.* 56(14)**,** 3867-3871. doi: 10.1002/anie.201612225.

Zhang, Y., Cheng, Y., Cai, H., He, S., Shan, Q., Zhao, H., et al. (2017). Catalyst-free aerobic oxidation of aldehydes into acids in water under mild conditions. *Green Chem.* 19(23)**,** 5708-5713. doi: 10.1039/C7GC02983G.
